# The role of macrophage polarization in ovarian cancer: from molecular mechanism to therapeutic potentials

**DOI:** 10.3389/fimmu.2025.1543096

**Published:** 2025-04-22

**Authors:** Chenchen Xu, Jiyu Chen, Mi Tan, Qingqing Tan

**Affiliations:** Department of Gynecology and Obstetrics, Changzhou Maternal and Child Health Care Hospital, Changzhou Medical Center, Nanjing Medical University, Changzhou, China

**Keywords:** ovarian cancer, tumor-associated macrophages, macrophage polarization, tumor microenvironment, therapy resistance, biomarkers, immunotherapy, extracellular vesicles

## Abstract

Ovarian cancer (OC) remains the most lethal gynecological malignancy, primarily due to its late-stage diagnosis, frequent recurrence, and resistance to conventional chemotherapy. A critical factor contributing to OC’s aggressiveness is the tumor microenvironment (TME), particularly the presence and polarization of tumor-associated macrophages (TAMs). TAMs, often skewed toward an immunosuppressive M2-like phenotype, facilitate tumor growth, angiogenesis, metastasis, and resistance to therapy. This comprehensive review delves into the multifaceted regulation of macrophage polarization in OC, highlighting key molecular pathways such as PTEN loss, Wnt/β-catenin signaling, NF-κB, Myc, STAT3, and JNK, among others. Additionally, it explores the role of chemokines, non-coding RNAs, and various proteins in modulating TAM phenotypes. Emerging evidence underscores the significance of extracellular vesicles (EVs) and ovarian cancer stem cells (CSCs) in promoting M2 polarization, thereby enhancing tumor progression and therapy resistance. The review also identifies critical biomarkers associated with macrophage polarization, including CD163, LILRB1, MUC2, and others, which hold prognostic and therapeutic potential. Therapeutic strategies targeting TAMs are extensively discussed, encompassing oncolytic viruses, engineered EVs, immunotherapies, nanoparticles, targeted therapies, and natural products. These approaches aim to reprogram TAMs from a pro-tumorigenic M2 state to an anti-tumorigenic M1 phenotype, thereby enhancing immune responses and overcoming resistance to treatments such as chemotherapy and immune checkpoint inhibitors. Furthermore, the review addresses the interplay between macrophage polarization and therapy resistance, emphasizing the need for novel interventions to modulate the TME effectively. By synthesizing current knowledge on macrophage polarization in ovarian cancer, this study underscores the potential of targeting TAMs to improve clinical outcomes and personalize treatment strategies for OC patients. Continued research in this domain is essential to develop robust therapeutic frameworks that can mitigate the immunosuppressive TME and enhance the efficacy of existing and novel cancer therapies.

## Introduction

Ovarian cancer (OC) remains the most lethal gynecological cancer, largely due to its late diagnosis, frequent recurrence, and resistance to chemotherapy (1). The complexity of the disease, characterized by its diverse histological subtypes and varying molecular features, presents challenges for effective treatment and prognosis (2). Standard treatment typically involves cytoreductive surgery followed by platinum-based chemotherapy, but novel approaches like PARP inhibitors, anti-angiogenic therapies, and drugs targeting the folate receptor alpha are showing potential, especially for patients with advanced or recurrent disease (3). Additional innovative treatments, such as immunotherapies, are being explored to improve outcomes. However, significant obstacles remain, including difficulties in early detection, overcoming drug resistance, and tailoring therapies to individual patients, emphasizing the need for further research to enhance survival rates and treatment personalization (4–6). Tumor-associated macrophages (TAMs) play a critical role in the immunosuppressive tumor microenvironment of ovarian cancers, which is a major reason why immunotherapies, such as immune checkpoint inhibitors, have limited success in treating this type of cancer (7, 8). A recent review has also highlighted the importance of TAMs in the ovarian cancer microenvironment, emphasizing their roles in tumor progression, chemoresistance, and immune modulation, further underscoring the significance of targeting TAMs in OC therapy (9). Targeting TAMs by reducing their recruitment, reprogramming them to an antitumor M1-like phenotype, or restoring their ability to phagocytose tumor cells can help reverse immunosuppression and improve the effectiveness of immunotherapies. Thus, TAMs are key therapeutic targets for enhancing the success of immunotherapy in ovarian cancer (10). Macrophage polarization refers to the process by which macrophages, versatile immune cells, adopt different functional states in response to signals from their environment. They can polarize into a pro-inflammatory (M1) state, which combats pathogens and promotes anti-tumor activity, or into an anti-inflammatory (M2) state, which supports tissue repair but can also aid tumor growth and metastasis (11). In cancer, TAMs are often skewed toward the M2-like phenotype due to signals from the tumor microenvironment (12). Notably, multiple studies have independently confirmed the critical role of the M1/M2 macrophage ratio in determining ovarian cancer prognosis and therapy response. For instance, analyses of tumor microenvironment composition in HGSOC have repeatedly demonstrated that higher M1/M2 ratios correlate with improved outcomes across different patient cohorts. Specifically, patients with high-grade serous ovarian cancer (HGSOC) exhibited a higher ratio of M1 macrophages (pro-inflammatory) to M2 macrophages (immunosuppressive), which was associated with longer OS, PFS, and platinum-free intervals (PFI). This positive correlation persisted regardless of the extent of cytoreductive surgery, indicating the importance of TAM polarization in patient outcomes. Additionally, patients with platinum-sensitive tumors had a higher M1/M2 ratio, suggesting that M1 polarization enhances chemotherapy effectiveness (13, 14). Analyzing 30 tissue samples from 24 ovarian cancer patients revealed that low-grade ovarian cancer had a higher M1/M2 ratio, suggesting a more immune-beneficial environment, while high-grade tumors, particularly in metastatic sites, showed increased M2 macrophage infiltration, indicating immune suppression. Treatment with platinum-based chemotherapy and bevacizumab increased M2 macrophages, especially in high-grade tumors, and also enlarged blood vessel diameters, correlating with more M2 infiltration (15). While this suggests a potential role for macrophage polarization in therapy resistance, larger patient cohorts are needed to validate these findings. These findings suggest that macrophage polarization plays a critical role in the development of malignant phenotypes in ovarian cancer cells, as well as in therapy resistance and disease progression. Targeting M2-polarized TAMs could, therefore, represent a promising therapeutic strategy to improve outcomes for ovarian cancer patients. Given the lack of specific reviews on this topic, this study aims to provide a comprehensive overview of the existing literature and findings related to macrophage polarization in ovarian cancer.

## Beyond the M1/M2 paradigm: macrophage diversity in ovarian cancer

Emerging evidence indicates that the macrophage landscape in ovarian cancer is highly heterogeneous, both across different patients (intertumoral heterogeneity) and within individual tumors (intratumoral heterogeneity). Single-cell RNA sequencing (scRNA-seq) and spatial transcriptomics have uncovered distinct TAM subpopulations that vary based on anatomical location, tumor subtype, and local microenvironmental factor (16). Notably, primary tumor sites often contain a mixture of M1 and M2 macrophages, while metastatic lesions—particularly within the omentum and ascitic fluid—exhibit a dominance of immunosuppressive M2-like TAMs (17). Macrophage-driven heterogeneity is further amplified by their interactions with tumor cells and stromal components. Tumor-derived extracellular vesicles (EVs) transport miRNAs such as miR-200b and miR-221-3p, which skew macrophages toward the M2 phenotype, reinforcing immune suppression and therapy resistance (18, 19). Additionally, metabolic factors such as lactate accumulation drive TAM polarization, contributing to spatially distinct macrophage phenotypes within the tumor microenvironment (20). This heterogeneity complicates therapeutic strategies targeting TAMs, as interventions need to account for the dynamic and location-specific nature of macrophage populations.

The traditional M1/M2 classification of macrophages, while foundational for understanding macrophage function, does not fully encompass the functional and transcriptional diversity of TAMs in ovarian cancer. Although M1 (pro-inflammatory) macrophages are associated with tumor suppression and M2 (anti-inflammatory) macrophages with tumor progression, recent research highlights that TAMs often exist along a broad and dynamic spectrum of activation states (21). Insights from scRNA-seq have revealed transcriptionally distinct and functionally diverse subpopulations of TAMs within ovarian tumors and their metastases, further supporting the notion of a continuum rather than a binary M1/M2 framework. For instance, some TAM subsets exhibit markers typical of both M1-like and M2-like phenotypes, performing seemingly contradictory roles within the TME. This emerging understanding challenges the oversimplified classification of TAMs and underscores their unique adaptability in response to the TME (22, 23)

The heterogeneity of TAM subtypes in ovarian cancer has been underscored by scRNA-seq studies that unveil multiple transcriptionally distinct populations. These studies have identified TAMs with immunosuppressive phenotypes, such as those marked by high expression of CD206 (a hallmark of M2-like macrophages), which co-exist with populations producing key pro-inflammatory cytokines like TNF-α. Furthermore, TAM heterogeneity is often spatially localized within distinct regions of a tumor or its metastatic niches (e.g., ascites fluid versus primary tumor sites) (16, 24). In cancer, TAMs are often skewed toward the M2-like phenotype due to signals from the tumor microenvironment. These M2-like TAMs play a critical role in cancer metastatic progression by promoting tumor survival, angiogenesis, immune suppression, and metastasis through the secretion of factors like IL-10, TGF-β, and VEGF (25). The presence of such heterogeneous subpopulations reflects the influence of diverse signaling cues in the tumor microenvironment, highlighting TAMs’ multifaceted and dynamic nature. Their roles extend to angiogenesis, immune exhaustion, stromal remodeling, and interactions with mesothelial cells during metastasis formation (16, 24). M2 phenotype macrophages are crucial in the progression of advanced epithelial ovarian cancer. Studies have revealed that a high density of CD163-positive M2 macrophages and a high CD163/CD68 ratio are significantly associated with worse progression-free survival (PFS) and overall survival (OS) in patients with advanced ovarian cancer (26). M2 macrophages, induced by IL-4, significantly enhance the proliferation, migration, and invasion of ovarian cancer cells while inhibiting their apoptosis *in vitro*. In contrast, M1 macrophages, show opposing effects, reducing tumor cell proliferation and promoting apoptosis. The co-culture of macrophages with ovarian cancer cells polarized macrophages toward the M2 phenotype, indicating that the tumor microenvironment favors the tumor-promoting M2 macrophages (27). In ovarian cancer, particularly in HGSOC, M1-like macrophages are significantly enriched in tumors with high homologous recombination deficiency (HRD), while M2-like macrophages are not (28). M1 macrophages, known for their anti-tumoral and pro-inflammatory functions, release cytokines like TNF-alpha and IL-2, promoting immune responses against the tumor. The selective enrichment of M1-like macrophages in HRD-high tumors suggests a more active immune environment, which could contribute to improved patient outcomes. This enrichment of M1 macrophages correlates with increased mutation burdens and neoantigen production in HRD-high tumors, further stimulating the immune response, while the absence of significant M2 macrophage activity highlights a less immunosuppressive environment in these cases (29).Altogether, these findings demonstrate the complex interplay between TAM subsets acting simultaneously to promote or restrain tumor development.

The polarization of TAMs is far more nuanced than previously thought, as various microenvironmental drivers shape their functional states. Cytokines such as IL-4, IL-10, and TGF-β have been identified as potent inducers of anti-inflammatory TAM polarization, whereas cytokines like IFN-γ and TNF-α favor pro-inflammatory phenotypes. Beyond cytokines, metabolic factors such as tumor-derived lactate drive TAM polarization toward immunosuppressive phenotypes by activating pathways like hypoxia-inducible factor 1-alpha (HIF-1α). Lipid metabolism, glutamine utilization, and mitochondrial oxidative phosphorylation are also known to contribute to macrophage reprogramming. Additionally, cellular interactions, including crosstalk with cancer-associated fibroblasts (CAFs) and T regulatory cells, further amplify TAM functional heterogeneity. These approaches expand upon the involvement of non-genetic alterations in TAMs as they dynamically shift their roles within the cancer ecosystem (30–32). Specific subsets of emerging TAM functional states defy the traditional M1/M2 paradigm entirely and exhibit unique characteristics. For instance, macrophages marked by high expression of TREM2 represent a distinct subpopulation implicated in immune suppression and tumor progression across cancers. These TAMs have been shown to promote immune evasion by attenuating T-cell activity within the TME (33). Likewise, tissue-resident macrophages that interact with stromal compartments and extracellular matrices display unique specialization, particularly within peritoneal metastases, as compared to their counterparts in primary tumor sites. Such adaptations underscore the highly plastic and context-dependent behavior of TAMs within different anatomical and functional regions of the tumor (34).

This dynamic diversity among TAMs has profound implications for therapeutic intervention. Strategies aimed at reprogramming TAMs or inhibiting their recruitment have achieved varying success depending on the specific subset or functional state being targeted. For example, agents targeting the CCL2/CCR2 axis have shown promise in reducing the recruitment of monocyte-derived TAMs, while inhibitors of colony-stimulating factor 1 receptor (CSF1R) blunt TAM support for tumor growth (35, 36). Recent research also highlights novel combinatorial approaches, such as targeting both CSF1R and TREM2 simultaneously, which may overcome immune suppression more effectively. Emerging therapies leveraging nanocarriers, exosome-based delivery systems, or chimeric antigen receptor (CAR)-based macrophages offer exciting avenues for developing precision therapeutics. These approaches recognize the importance of subset-specific targeting to maximize therapeutic efficacy and minimize off-target immune effects in ovarian cancer treatment (37).

By shifting the focus from the classical M1/M2 paradigm to a more comprehensive view of TAM heterogeneity, researchers can better understand the transcriptional, functional, and metabolic diversity within the ovarian cancer TME. This updated classification framework provides a clearer foundation for designing therapies tailored to specific TAM subsets and microenvironmental factors. Through precision targeting of TAMs, it may be possible to optimize therapeutic approaches against immunosuppressive and tumor-promoting macrophage subpopulations, thereby improving clinical outcomes for patients with ovarian cancer. The incorporation of single-cell technologies, such as scRNA-seq, paves the way for unraveling further complexities of macrophage biology in cancer.

## Regulators of macrophage polarization in ovarian cancer

Macrophage polarization in cancer involves two main pathways leading to pro-inflammatory M1-like and anti-inflammatory M2-like phenotypes. M1 polarization, which promotes tumor suppression, is driven by IFN-γ, TNF-α, and TLR signaling through pathways like JAK/STAT1, NF-κB, and MAPKs (JNK, ERK), resulting in the production of pro-inflammatory cytokines and reactive oxygen species. In contrast, M2 polarization, associated with tumor promotion, is activated by signals like IL-4, IL-10, and TGF-β, primarily through the JAK/STAT6, PI3K/AKT/mTOR, and TGF-β/SMAD pathways, promoting tissue repair, immune suppression, and angiogenesis. Dual-role pathways such as NF-κB and ERK can contribute to either polarization depending on the context. Targeting these pathways therapeutically aims to shift macrophages from a tumor-promoting M2-like state to a tumor-suppressing M1-like state (38). This section reviews the pathways that interact with macrophage polarization in ovarian cancer.

### Intrinsic tumor signaling pathways influencing macrophage polarization

#### PTEN

**Figure 1:**
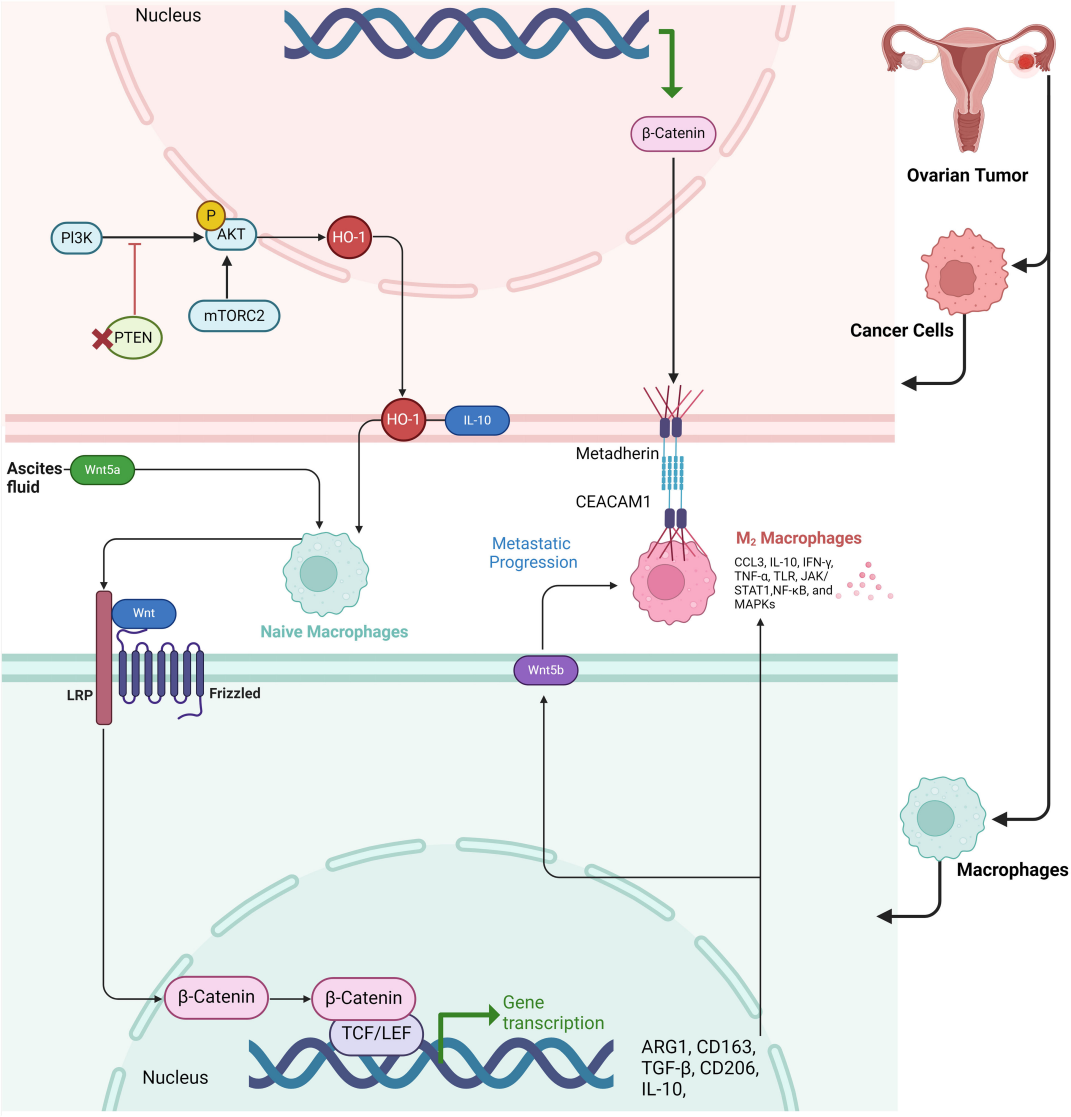
Schematic representation of the Wnt and PI3K/AKT pathways involved in macrophage polarization and metastatic progression in the tumor microenvironment of ovarian cancer. The figure illustrates how cancer cells in ovarian tumors release factors into the ascites fluid, including Wnt5a, which binds to Frizzled receptors on naïve macrophages, activating β-catenin signaling and promoting a shift toward an M2 macrophage phenotype. In the tumor cells, the PI3K/AKT/mTORC2 pathway upregulates HO-1, leading to IL-10 production, which further aids in immune modulation and supports the M2 macrophage polarization. The M2 macrophages, characterized by high expression of markers such as ARG1, CD163, TGF-β, CD206, and IL-10, facilitate metastatic progression by promoting cellular interactions via Metadherin and CEACAM1 receptors. The transcriptional activation of β-catenin in both macrophages and cancer cells enhances the transcription of genes associated with immune suppression and metastasis. Key signaling molecules in the M2 macrophages, including CCL3, IL-10, IFN-γ, and NF-κB, contribute to the tumor-promoting environment.

PTEN loss in HGSOC significantly impacts macrophage dynamics, shaping the tumor microenvironment. PTEN deficiency activates the PI3K signaling pathway, which promotes the expansion of a specific population of resident-like macrophages in omental tumors (39). These macrophages express high levels of the enzyme heme oxygenase-1 (HMOX1), which contributes to tumor growth and correlates with poor survival outcomes. PTEN loss drives the recruitment of these HMOX1-high macrophages from peritoneal fluid macrophages rather than from monocyte-derived macrophages. These resident macrophages exhibit immunosuppressive traits, aiding tumor progression by creating a less favorable microenvironment for adaptive immune responses. Moreover, human HGSOC tumors also show a similar enrichment of HMOX1-high macrophages, further linking PI3K/mTOR pathway activation and poor prognosis. Targeting these macrophages or inhibiting HMOX1 has shown potential therapeutic value in reducing tumor growth and improving survival outcomes in preclinical models (40)​. PTEN deficiency in ovarian cancer leads to the recruitment and polarization of macrophages into an M2-like phenotype, which is associated with an immunosuppressive tumor microenvironment. These M2-like macrophages promote tumor progression by supporting immune evasion and reducing cytotoxic T-cell activity. PTEN-deficient tumors show increased infiltration of these M2 macrophages in the ascites and tumor stroma, contributing to more aggressive tumor behavior and decreased responsiveness to chemotherapy. Additionally, PTEN deficiency also elevates levels of immunosuppressive cytokines, such as IL-10, further reinforcing the M2-like suppressive macrophage dominance (**Figure 1**) (41).

#### Wnt/β-catenin signaling

The Wnt/β-catenin signaling pathway is essential for promoting macrophage M2 polarization, which plays a significant role in ovarian cancer progression (42). Activation of this pathway leads to the nuclear translocation of β-catenin, which drives the reprogramming of macrophages from an M0 to an M2 phenotype. This polarization supports tumor growth and metastasis by enhancing cancer cell migration and invasion. Inhibiting β-catenin nuclear translocation can reverse M2 polarization, reducing its tumor-promoting effects, indicating the pathway’s critical role in ovarian cancer metastasis (43) (44). Paracrine WNT signaling loops between M2-like macrophages and ovarian cancer stem cells (CSCs) create a reciprocal interaction that promotes tumor progression. In ovarian cancer, CSCs activate macrophages, driving them to adopt an immunosuppressive M2-like phenotype, characterized by increased CD206 expression and IL-10 secretion. These macrophages, in turn, release WNT ligands, particularly WNT5B, which further enhance CSC stemness by maintaining high levels of ALDH+ cells and promoting CSC chemoresistance and invasiveness. This mutual signaling between CSCs and M2 macrophages, facilitated by WNT pathways, strengthens pro-tumoral activities, forming a feedback loop that exacerbates tumor aggressiveness and resistance to treatment. Targeting this paracrine WNT signaling could offer therapeutic opportunities to disrupt the tumor-supportive microenvironment (45). Host-derived Wnt5a, secreted by peritoneal mesothelial cells and adipocytes within the ovarian cancer microenvironment, plays a critical role in promoting ovarian cancer metastasis. High levels of Wnt5a in ascites fluid enhance the adhesion, migration, and invasion of ovarian cancer cells, driving their colonization of the peritoneum (46, 47). Wnt5a induces these prometastatic behaviors by activating the Src family kinase Fgr, which in turn accelerates cellular motility and adhesion, critical steps in metastatic progression. In addition to directly influencing cancer cell behavior, Wnt5a also shapes an immunosuppressive tumor microenvironment by skewing immune cell profiles, favoring the presence of M2 macrophages and regulatory T cells while reducing cytotoxic T cells and M1 macrophages. This results in a tumor-friendly immune landscape that further promotes metastasis. Knockout of Wnt5a in host cells dramatically reduces tumor burden and alters immune infiltration patterns, suggesting that targeting Wnt5a or its downstream effector, Fgr, may be an effective therapeutic strategy for halting ovarian cancer metastasis (46). The β-catenin-metadherin/CEACAM1-CCL3 axis plays a critical role in mediating metastatic heterogeneity by orchestrating interactions between metastatic tumor cells and TAMs. In highly metastatic (HM) ovarian cancer cells, the β-catenin signaling pathway is upregulated, leading to an increasing the expression of metadherin on the tumor cell surface. Metadherin engages with CEACAM1, a receptor on TAMs, triggering the production of CCL3, a chemokine that promotes macrophage recruitment and retention at metastatic sites. This interaction creates a positive feedback loop, wherein HM cells polarize macrophages to a pro-tumor TAM phenotype. Upon contact with macrophages, a subset of HM cells undergoes polyploidization, a process where cells fail to complete cell division, resulting in larger, multinucleated cells. These polyploid cells are more aggressive, migratory, and resistant to therapy, contributing to tumor heterogeneity and metastasis (**Figure 1**) (48).

#### NF-KB pathway

Enhanced canonical NF-kappaB signaling in macrophages is enough to reduce tumor progression in syngeneic mouse models of ovarian cancer by fostering an anti-tumor immune environment (49). In a transgenic mouse model (IKFM) with inducible NF-kappaB activation in macrophages, researchers found that TAMs were reprogrammed from a pro-tumor M2 state to an anti-tumor M1 state. This reprogramming was linked to increased tumor necrosis and greater infiltration of immune cells, particularly cytotoxic CD8+ T cells, into both solid tumors and ascitic fluid. Additionally, these macrophage changes elevated the levels of the chemokine CXCL9, which attracts CD8+ T cells and further boosts the anti-tumor immune response. These effects were observed in both established tumors and early tumor growth, indicating that NF-kappaB activation in TAMs creates a more immunogenic tumor microenvironment, slowing tumor growth. This suggests that targeting macrophage NF-kappaB signaling could provide a promising new approach for improving immune-based treatments in ovarian cancer (50). Ovarian cancer stem cells (OCSCs) induce the M2 polarization of macrophages primarily through the activation of the PPARγ pathway and the suppression of the NF-κB pathway. When co-cultured with macrophages, OCSCs increase the expression of M2-associated markers such as the mannose receptor (MR), interleukin-10 (IL-10), and arginase-1 (Arg-1), while simultaneously reducing the expression of M1 markers like tumor necrosis factor-α (TNF-α), inducible nitric oxide synthase (iNOS), and CD86. This shift toward the M2 phenotype, which supports tumor growth and immune suppression, is driven by the upregulation of PPARγ, a nuclear receptor involved in anti-inflammatory responses. The suppression of NF-κB, a key transcription factor associated with inflammatory responses, further reinforces this polarization. Inhibition of PPARγ using a specific antagonist (GW9662) reverses the effects of OCSCs, restoring NF-κB activity and promoting M1 polarization. This indicates that OCSCs exploit the PPARγ/NF-κB signaling axis to skew macrophages toward a tumor-promoting M2 phenotype (51). In contrast, pro-inflammatory M1 macrophages enhance the metastatic potential of ovarian cancer cells primarily by activating the NF-κB signaling pathway. While preclinical models strongly suggest that NF-κB activation in macrophages enhances anti-tumor immunity, caution is needed when extrapolating these findings to human studies (52). Notably, studies in breast and colorectal cancer have established CXCL1 as a driver of EMT, yet direct evidence for this mechanism in ovarian cancer remains limited, warranting further validation in larger clinical datasets Secreted factors from M1 macrophages, including TNF-α and CXCL1, have been shown in other cancers such as breast and colorectal cancer to promote cancer cell migration and invasion via NF-κB activation. CXCL1, which is highly secreted by TAMs in breast cancer, has been identified as a key driver of epithelial–mesenchymal transition (EMT), increasing cancer cell motility (53). Mechanistic studies suggest that CXCL1 directly binds to the SOX4 promoter, activating its transcription via NF-κB signaling, ultimately promoting tumor progression and metastasis. Similarly, CXCL1 overexpression in colorectal cancer has been linked to enhanced tumor angiogenesis and recruitment of M2-like TAMs, further supporting an immunosuppressive tumor microenvironment (54). In ovarian cancer, the involvement of CXCL1 in NF-κB-driven metastasis remains underexplored, but these findings suggest a potential role for CXCL1 in regulating macrophage-cancer cell interactions within the ovarian tumor microenvironment. Blocking CXCL1 or its downstream NF-κB activation may provide a novel therapeutic avenue for limiting ovarian cancer metastasis.

Another key example of this NF-κB-driven interplay between ovarian cancer cells, macrophages, and the tumor microenvironment is seen with periostin (POSTN), a matrix protein overexpressed in highly invasive ovarian cancer cells (55). POSTN has been identified as a crucial mediator of macrophage recruitment and polarization through integrin-ERK-NF-κB signaling. In ovarian cancer, POSTN enhances integrin β3 and β5 signaling, which activates ERK and NF-κB pathways in tumor cells. This results in the secretion of macrophage-attracting cytokines such as MIP-1β, MCP-1, TNF-α, and RANTES, leading to increased chemotaxis of monocytes and their subsequent polarization into immunosuppressive M2 macrophages. Notably, tumors overexpressing POSTN harbored more TAMs and exhibited greater metastatic potential, further emphasizing the role of NF-κB signaling in shaping an aggressive tumor microenvironment. Additionally, POSTN-induced NF-κB activation stimulates the production of TGF-β2, which drives the differentiation of adipose-derived stromal cells into cancer-associated fibroblasts (CAFs)—another key player in ovarian cancer progression. Clinically, high POSTN expression correlates with advanced-stage disease and poor patient survival, underscoring its potential as both a biomarker and a therapeutic target (56)​. Together, these findings highlight the paradoxical role of NF-κB signaling in ovarian cancer. While its activation in TAMs can reprogram them toward an anti-tumor M1 phenotype, its activation in ovarian cancer cells—via CXCL1, MIP-1β, MCP-1, TNF-α, and RANTES, or other secreted factors might be involved in driving metastasis, immune suppression, and therapy resistance. Understanding this dual role is critical for developing targeted therapies that selectively inhibit NF-κB-driven tumor progression while preserving its anti-tumor immune functions.

**Figure 2:**
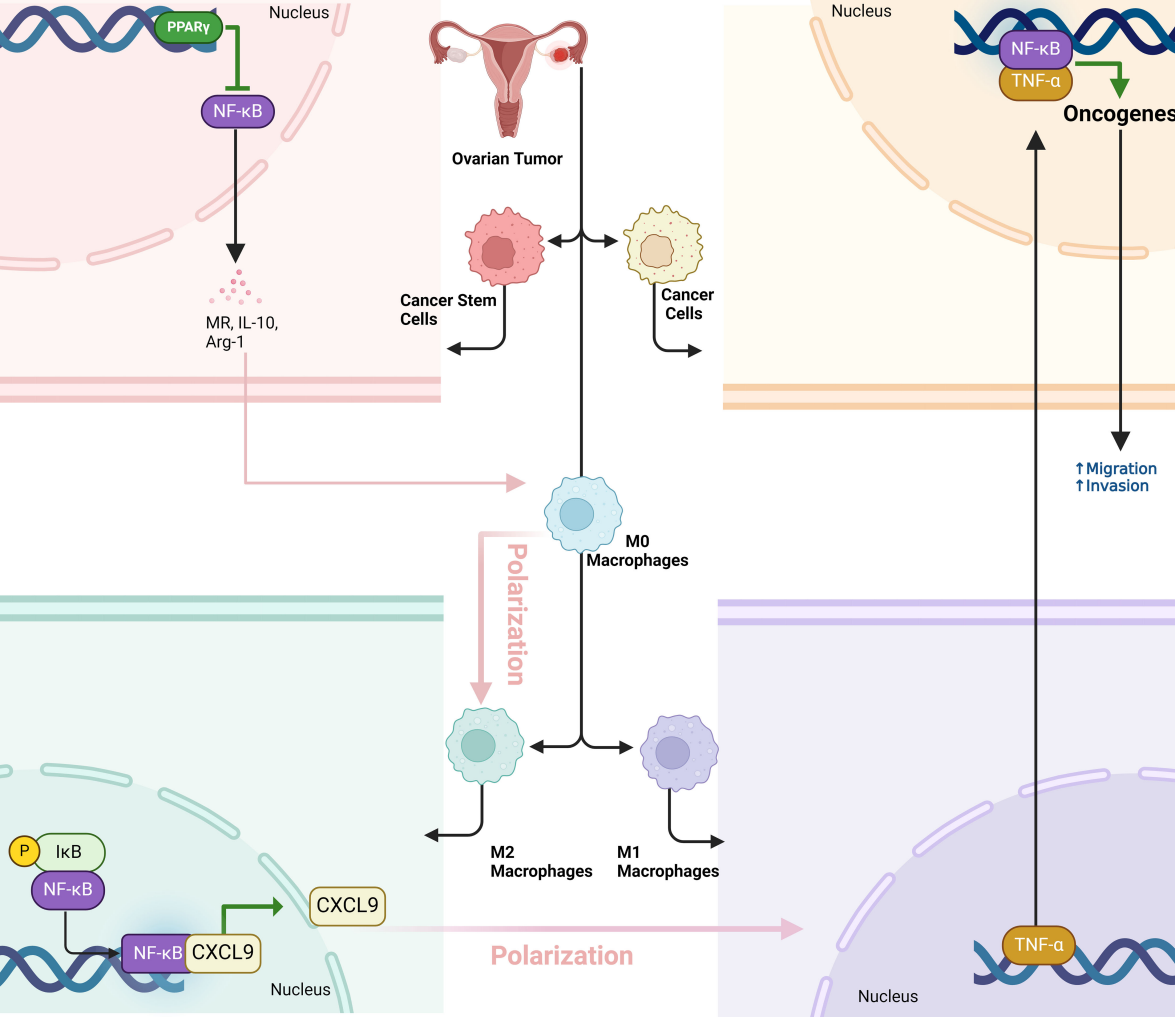
The dual role of NF-κB signaling in macrophage polarization and ovarian tumor progression. Cancer stem cells (OCSCs) promote a pro-tumor M2 macrophage phenotype by activating PPARγ and inhibiting NF-κB, which supports tumor growth. Conversely, NF-κB activation in macrophages reprograms them into an anti-tumor M1 phenotype, boosting immune cell infiltration (especially CD8+ T cells) and tumor necrosis. However, M1 macrophages can also enhance tumor metastasis by releasing TNF-α, which activates NF-κB in cancer cells, promoting migration and invasion. This highlights NF-κB’s dual role in tumor immunity and metastasis.

When ovarian cancer cells are exposed to M1 macrophage-conditioned media, their migration and invasion abilities significantly increase, which are key factors in metastasis (14). M1 macrophages release pro-inflammatory cytokines, particularly TNF-α, which triggers the nuclear translocation of NF-κB subunits (p50, p65) from the cytosol to the nucleus in ovarian cancer cells. This translocation leads to an increase in NF-κB’s transcriptional activity, which drives the expression of genes that promote cancer cell motility and invasiveness. The use of an NF-κB inhibitor (TPCK) or NF-κB-specific siRNA reduced this metastatic potential, confirming that NF-κB activation is crucial for M1 macrophage-induced metastasis. Additionally, co-treatment with a TNF-α inhibitor (etanercept) reversed NF-κB activation, further demonstrating that TNF-α from M1 macrophages is the primary mediator of this process (**Figure 2**) (57).

#### STAT signaling

**Figure 3:**
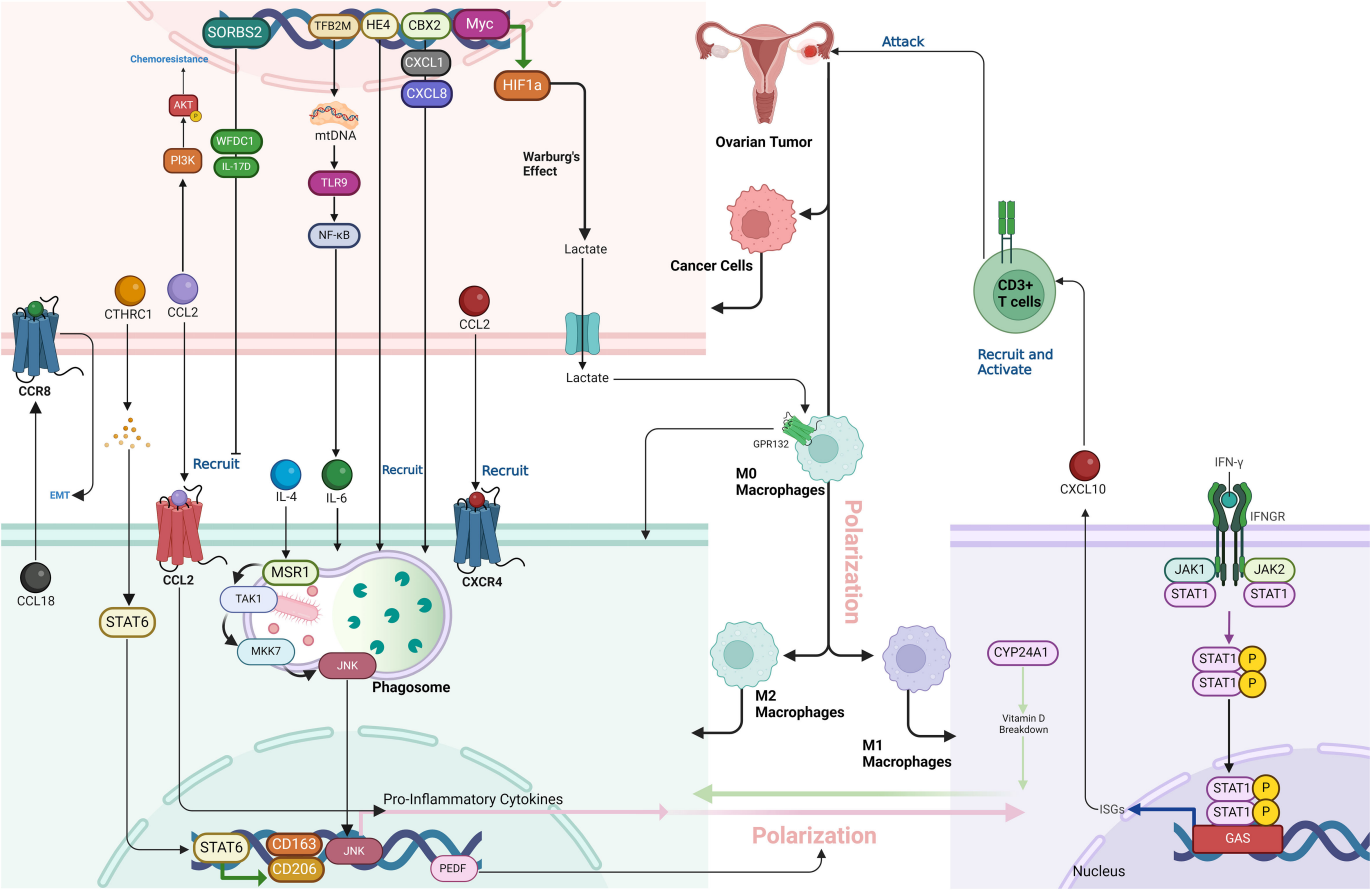
The complex interactions within the tumor microenvironment (TME) of ovarian cancer, emphasizing the role of key proteins, including Myc, HIF1a, GPR132, CTHRC1, STAT6, CCL2, CXCL8, CXCL10, IL-6, MSR1, TLR9, NF-κB, CYP24A1, CD206, CD163, HE4, WFDC1, IL-17D, TFB2M, PEDF, and JNK, in promoting an immunosuppressive and pro-tumorigenic landscape. These proteins collectively drive macrophage polarization, immune cell recruitment, and CD8+ T cell suppression, facilitating tumor progression and resistance to therapies in ovarian cancer.

Alternatively activated macrophages (AAMs) promote the metastasis of ovarian cancer by secreting factors like FLT3L, leptin, and HB-EGF, which stimulate the spreading of HGSOC spheroids across the extracellular matrix (ECM). Each cancer cell line responds to different AAM-derived factors, but they all funnel through a common JAK2/STAT3 signaling pathway. Activation of this pathway leads to increased secretion of MMP-9, a matrix-degrading enzyme, which facilitates spheroid disaggregation and migration. This mechanism highlights JAK2/STAT3 as a central mediator of cancer cell spreading, presenting a potential therapeutic target to inhibit ovarian cancer metastasis (58). In ovarian cancer tissues, CTHRC1 is overexpressed, especially in advanced-stage tumors, and is strongly correlated with increased infiltration of M2-like TAMs, which are known to facilitate tumor growth and metastasis (59). CTHRC1 is a secreted protein that, once released into the tumor microenvironment, influences the immune landscape by activating the STAT6 pathway in macrophages (60). This activation leads to the phosphorylation of STAT6, a critical step in the polarization of macrophages toward the M2 phenotype, characterized by the expression of markers like CD206 and CD163. These M2-like TAMs, in turn, promote cancer cell migration and invasion, further contributing to tumor progression. CTHRC1-driven macrophage polarization occurs in a dose-dependent manner, both *in vitro* and *in vivo*, with higher CTHRC1 levels leading to greater activation of the STAT6 pathway and stronger M2 polarization. This indicates that CTHRC1 not only recruits macrophages but also reprograms them into an immunosuppressive M2 state, which supports cancer metastasis, making CTHRC1 a potential therapeutic target for disrupting this pro-tumorigenic interaction in ovarian cancer (59). The co-culture of ovarian cancer stem-like cells (OCSLCs) with macrophages derived from THP-1 cells promoted stemness in SKOV3 ovarian cancer cells through the IL-8/STAT3 signaling pathway. This interaction led to the polarization of macrophages into the tumor-promoting M2 phenotype, characterized by increased secretion of IL-10, VEGF, and MMP-9, alongside reduced levels of IL-12 and NO. IL-8, produced during co-culture, activated STAT3 in macrophages, which in turn enhanced the stemness of SKOV3 cells by increasing their ability to form spheres and colonies, and by upregulating cancer stem cell markers CD133 and CD44. Blocking IL-8 or inhibiting STAT3 activation disrupted these effects, indicating that the IL-8/STAT3 axis plays a crucial role in the interaction between OCSLCs and macrophages, driving tumor progression and cancer cell stemness (**Figure 3**) (61).

#### JNK signaling

In ovarian cancer, JNK signaling is crucial in driving macrophage polarization from an anti-inflammatory M2 state to a pro-inflammatory state (62). Triggering the macrophage scavenger receptor 1 (MSR1) in IL-4-activated M2 macrophages induces K63-linked polyubiquitylation, which recruits the TAK1/MKK7/JNK signaling complex to the phagosome. Activation of JNK leads to the production of pro-inflammatory cytokines and a shift in macrophage phenotype. This process is observed in TAMs within ovarian cancer tissue, where elevated MSR1 ubiquitylation and JNK activation contribute to the inflammatory environment, potentially promoting tumor progression and metastasis. This signaling pathway highlights the importance of macrophage plasticity in cancer progression and suggests JNK as a potential therapeutic target (**Figure 3**) (63).

### Chemokines and cytokines

Chemokines and their receptors play a pivotal role in the polarization of macrophages by influencing their recruitment, differentiation, and activation in response to various environmental cues (64). These small signaling proteins guide macrophages to specific tissues where they adopt different functional phenotypes based on the local microenvironment. For instance, chemokine receptors like CCR2, CCR5, and CXCR4 help recruit monocytes to inflammation or tissue damage sites, where they differentiate into macrophages. Chemokines such as CCL2, CCL18, and CXCL12 are instrumental in promoting either a pro-inflammatory M1 phenotype, which is associated with pathogen clearance and tissue damage, or an anti-inflammatory M2 phenotype, which is involved in tissue repair, fibrosis, and tumor progression (65). These chemokine-mediated signaling pathways not only determine the type of macrophage polarization but also contribute to the macrophages’ specific roles in diseases such as cancer, atherosclerosis, fibrosis, and metabolic disorders. Thus, chemokines are crucial regulators of macrophage plasticity and their function in immune responses (66). A study highlighted the critical role of the CCL2-CCR2 axis in mediating paclitaxel resistance in ovarian cancer cells through both autocrine and paracrine signaling mechanisms. Paclitaxel-resistant ovarian cancer cells were shown to overexpress CCL2, which not only promoted chemoresistance via PI3K/Akt and NF-κB signaling pathways but also attracted TAMs. These TAMs, once recruited by the CCL2 secreted by resistant cancer cells, were polarized into an M2 phenotype, which is known to promote tumor progression and further enhance resistance to paclitaxel. Inhibition of the CCL2/CCR2 axis using a CCR2 inhibitor significantly increased paclitaxel sensitivity in both *in vitro* and *in vivo* models, suggesting that targeting this signaling pathway could be a promising therapeutic strategy to overcome chemoresistance in ovarian cancer. This dual mechanism involving both the tumor cells’ autocrine signaling and macrophage recruitment contributes to the poor therapeutic response and highlights the potential benefit of CCR2 inhibitors in treating ovarian cancer (35). In ovarian cancer, CXCR2 is significantly associated with macrophage infiltration, particularly influencing both M1 and M2 macrophages in the tumor microenvironment. CXCR2 expression negatively correlates with the presence of both M1 and M2 macrophages, indicating its role in reducing macrophage infiltration. This suggests that CXCR2 may contribute to creating an immunosuppressive tumor environment by limiting macrophage activity, particularly M1 macrophages, which are typically involved in anti-tumor immune responses, and M2 macrophages, which are often associated with promoting tumor growth and immunosuppression (67). CXCL10 is crucial in M1-polarized TAMs in ovarian cancer by promoting antitumor immunity. M1-polarized TAMs, particularly in HGSOC, secrete CXCL10 in response to IFNγ signaling, which attracts T-cells into the tumor microenvironment. This enhances the immune response against the tumor, as CXCL10 helps to recruit and activate CD3+ T-cells, facilitating a coordinated attack on cancer cells. The presence of CXCL10+ M1-type TAMs is associated with better clinical outcomes, greater chemotherapy response, and improved survival in ovarian cancer patients. Multiple studies have confirmed this association in HGSOC cohorts, demonstrating a strong correlation between CXCL10 expression and improved immune infiltration. In contrast, ovarian cancer subtypes lacking CXCL10+ TAMs, such as clear cell carcinoma (CCC), exhibit poorer immune infiltration and worse prognoses. Therefore, CXCL10 in M1-polarized TAMs is a key factor in driving immune-mediated tumor suppression and enhancing therapeutic efficacy in ovarian cancer. This highlights the need for subtype-specific investigations before generalizing the role of CXCL10 across all ovarian cancer cases (68, 69). The CXCL12-CXCR4 axis in ovarian tumors promotes the recruitment and retention of M2 macrophages. This axis inhibits the presence of M1 macrophages, which are essential for antitumor immunity due to their pro-inflammatory and immune-activating properties. By blocking CXCL12-CXCR4, for example, using the antagonist AMD3100, the accumulation of M2 macrophages is reduced, leading to a shift toward M1 macrophage polarization. This shift enhances the tumor’s immune response by activating cytotoxic T cells and producing pro-inflammatory cytokines, creating a less favorable environment for tumor survival. In combination with immune checkpoint inhibitors like anti-PD-1, blocking this pathway further strengthens antitumor immune activity by facilitating M2-to-M1 macrophage reprogramming (70). M2-TAMs promote the EMT of ovarian cancer (OvCa) cells within tumor spheroids by secreting chemokine (C-C motif) ligand 18 (CCL18). CCL18 binds to its receptor, CCR8, on OvCa cells, triggering the activation of the EMT process, which transforms these cells from an epithelial to a mesenchymal state, characterized by increased invasiveness and migratory abilities. This transition is mediated by the upregulation of EMT-inducing transcription factors like ZEB1. ZEB1 not only drives EMT but also stimulates the production of macrophage colony-stimulating factor (M-CSF) in OvCa cells, which further polarizes TAMs into the pro-tumorigenic M2 subtype, creating a feedback loop that enhances the aggressiveness of OvCa spheroids and promotes metastasis (**Figure 3**) (71).

Recent findings by Mollaoglu et al. provide crucial insights into the role of IL-4 in shaping specific macrophage subsets in ovarian cancer. This study identified IL-4 as a key tumor-derived factor that drives macrophage polarization, particularly promoting the MARCO-expressing macrophage subset in the ovarian tumor microenvironment. These macrophages exhibit an immunosuppressive phenotype, contributing to tumor progression and resistance to immune checkpoint blockade (ICB). The study further demonstrated that IL-4 signaling operates within distinct TME neighborhoods, where even a small fraction of IL-4-producing ovarian cancer cells can exert a significant impact on the surrounding immune landscape. IL-4-mediated macrophage programming was found to be spatially restricted, emphasizing the localized effects of cytokine gradients within the tumor. These findings underscore the complexity of macrophage polarization beyond the traditional M1/M2 dichotomy, revealing that distinct factors, such as IL-4, drive specific gene expression programs within macrophage subsets that have unique immunosuppressive functions in ovarian cancer (72). IL-4, IL-10 and TGF-β are also central to macrophage-mediated immunosuppression in ovarian cancer (68). IL-10 suppresses pro-inflammatory signaling and supports the expression of CD163 and CD206, hallmark M2 markers associated with poor prognosis. TGF-β further enhances the immunosuppressive function of macrophages by promoting extracellular matrix remodeling, angiogenesis, and immune escape mechanisms (73). Conversely, pro-inflammatory cytokines such as IFN-γ and TNF-α drive M1 polarization, enhancing tumor immunity (70). IFN-γ, secreted by activated CD8+ T cells and NK cells, induces the production of CXCL10, a key chemokine that recruits additional effector T cells into the TME. High levels of CXCL10+ TAMs have been correlated with improved chemotherapy response and overall survival in ovarian cancer patients, particularly in HGSOC (68).

Collectively, these findings underscore the complex interplay between chemokines, cytokines, and macrophages in ovarian cancer. While immunosuppressive factors such as CCL2, CXCL12, IL-4, and IL-10 sustain an immune-excluded TME, pro-inflammatory mediators like IFN-γ, TNF-α, and CXCL10 counteract these effects by promoting immune cell infiltration and tumor suppression. Targeting the balance between these opposing forces offers a promising strategy for modulating macrophage polarization and enhancing the efficacy of ovarian cancer therapies.

### Epigenetic and transcriptional regulators of macrophage polarization

#### Myc

Myc-mediated inhibition of HIF1a degradation plays a key role in fostering an immunosuppressive environment in ovarian cancer by affecting both macrophage polarization and CD8 T cell activity (74). Overexpression of Myc stabilizes HIF1a, a transcription factor that drives the Warburg effect, leading to increased lactic acid production in tumor cells (75). This lactic acid acts as a signaling molecule, inducing macrophages to polarize into the M2 phenotype, which is known for its immunosuppressive properties. This process is dependent on the activation of Gpr132, a receptor on macrophages that is activated by the elevated lactic acid levels. Once polarized, M2 macrophages release factors that inhibit CD8 T cell proliferation, lower IFN-γ secretion, and weaken the CD8 T cells’ ability to target and kill tumor cells. Thus, Myc not only promotes metabolic changes via HIF1a stabilization but also establishes an immune-suppressive tumor microenvironment by enhancing M2 macrophage polarization and directly impairing CD8 T cell function. This dual effect allows the tumor to evade immune surveillance and promotes its progression, contributing to the challenges in treating ovarian cancer (**Figure 3**) (74).

#### CBX2

Chromobox 2 (CBX2) is an epigenetic regulator and a component of polycomb repressor complex 1 (PRC1), known for its role in chromatin modification and gene transcription (76). In the context of HGSOC, CBX2 is overexpressed in the majority of cases and is associated with poor prognosis and chemotherapy resistance (77). Its primary role in the tumor immune microenvironment (TIME) is to drive an immunosuppressive state by promoting the recruitment and polarization of macrophages toward a tumor-promoting M2-like phenotype. This polarization leads to decreased immune surveillance and increased tumor progression. CBX2 also regulates the expression of immune-modulatory genes, including cytokines such as CXCL1 and CXCL8, which further enhance the recruitment of TAMs. Thus, CBX2 plays a crucial role in remodeling the TIME to support tumor growth and therapy resistance, making it a potential therapeutic target to improve immune responses against tumors (**Figure 3**) (78).

#### GATA3

**Figure 4:**
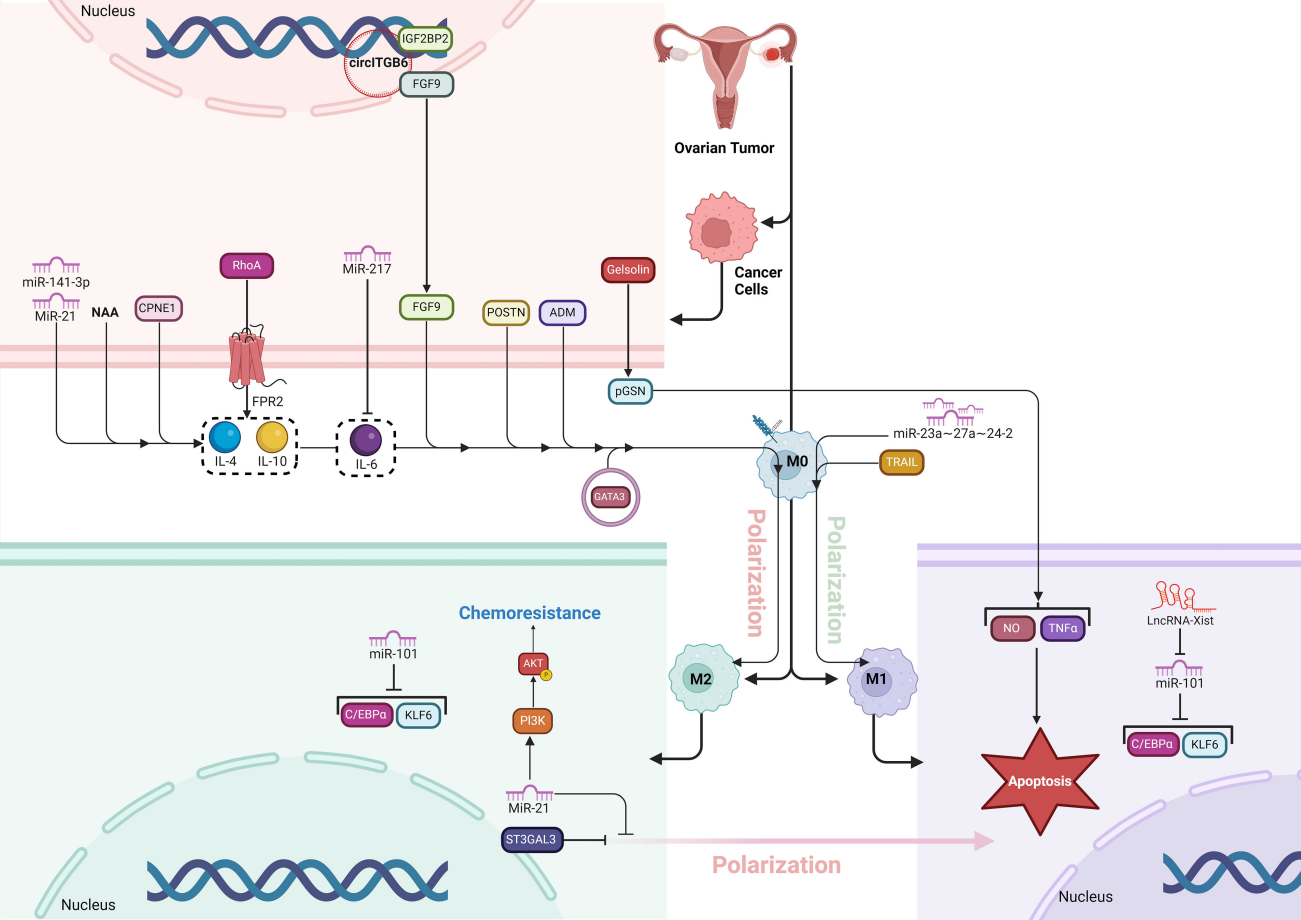
The figure illustrates how various molecular factors drive TAM polarization and chemoresistance in the OC microenvironment. pGSN, secreted by OC cells, promotes chemoresistance by inhibiting M1 macrophages’ anti-tumor functions and favoring M2 dominance. FPR2 activates RhoA, enhancing cell migration and fostering an immunosuppressive M2-supporting environment. GATA3, highly expressed in HGSOC, promotes M2 polarization, aiding tumor growth and chemoresistance. St3gal3 supports immune evasion by increasing M2 macrophages, while ADM drives M2 polarization through RhoA signaling. TRAIL, on the other hand, induces M1 polarization, boosting anti-tumor immunity. CPNE1 and POSTN further increase M2 prevalence, supporting tumor progression. Various ncRNAs (miR-21, miR-141-3p, miR-217, LncRNA-Xist) and circITGB6 regulate polarization and chemoresistance by targeting key pathways, with miR-21 enhancing M2-related chemoresistance via PI3K/AKT. These factors collectively establish a tumor-promoting environment, revealing potential therapeutic targets to improve OC treatment.

GATA3 is a transcription factor that plays a pivotal role in immune cell differentiation and function, and it has emerged as a master regulator in the interaction between TAMs and high-grade serous ovarian carcinoma (HGSOC) (79). In HGSOC, GATA3 is highly expressed and contributes to poor clinical outcomes by promoting tumor proliferation, migration, angiogenesis, and resistance to chemotherapy, particularly in cases with mutant TP53 (80). GATA3 is abundantly released via exosomes, where it drives the polarization of macrophages to the pro-tumorigenic M2 phenotype, enhancing tumor growth and immune evasion. Moreover, GATA3 orchestrates complex cross-talk between TAMs and tumor cells, facilitating epithelial-mesenchymal transition (EMT), chemoresistance, and overall tumor progression. Targeting GATA3 has shown potential to impair these tumor-supporting interactions, making it a promising therapeutic target for treating HGSOC (**Figure 4**) (79).

#### St3gal3

St3gal3 is an enzyme responsible for catalyzing the synthesis of α2,3-linked sialic acids, which play a crucial role in promoting immune evasion and tumor progression in ovarian cancer (81). Elevated St3gal3 levels are associated with poor prognosis in HGSOC by contributing to an immunosuppressive TME characterized by increased infiltration of pro-tumor macrophages and reduced CD8^+^ T-cell activity. Knockdown or inhibition of St3gal3 reprograms this environment by boosting immune cell infiltration, enhancing the presence of cytotoxic CD8+ T cells, and repolarizing TAMs from a tumor-promoting M2-like phenotype to an antitumor M1-like state. Additionally, blocking sialylation with pharmacological agents like ambroxol sensitizes the tumor to immune checkpoint blockade (ICB) therapies, such as anti-PD-1 and anti-CTLA4, significantly improving tumor control and extending survival in preclinical models. Thus, targeting St3gal3 and sialylation offers a promising strategy to enhance the effectiveness of immunotherapy in ovarian cancer (**Figure 4**) (82).

#### Non-coding RNAs

Non-coding RNAs (ncRNAs), including microRNAs (miRNAs) and long non-coding RNAs (lncRNAs), play crucial roles in regulating macrophage polarization, a process that impacts tumor progression and immune response. NcRNAs influence this polarization by targeting key genes and pathways that either promote M1 or M2 phenotypes (83). For instance, some miRNAs like miR-16 and miR-155 drive M1 polarization, enhancing anti-tumor activity, while others like miR-145 and miR-21 promote M2 polarization, supporting tumor growth and metastasis. Similarly, lncRNAs like GAS5 favor M1 polarization, while others like NEAT1 and MALAT1 promote M2 macrophages in some cancers. By regulating these polarization states, ncRNAs are central to shaping the tumor microenvironment, offering potential therapeutic targets to modulate immune responses and combat cancer (84). MiR-21 has a key role in the polarization of macrophages, particularly by promoting the M2 macrophage phenotype. In ovarian cancer, M2 macrophages, characterized by anti-inflammatory properties, enhance the chemoresistance of cancer cells. MiR-21 influences this process by upregulating M2 markers (like CD206 and IL-10) and downregulating M1 markers (such as TNF-α and iNOS), thus driving macrophages toward the tumor-promoting M2 phenotype. The presence of M2 macrophages increases the resistance of ovarian cancer cells to chemotherapy, particularly cisplatin. This chemoresistance is mediated through the PI3K/AKT signaling pathway, where M2 macrophages with elevated miR-21 levels enhance the survival and drug resistance of ovarian cancer cells. Conversely, inhibiting miR-21 can reduce this chemoresistance by repolarizing M2 macrophages back to the M1 phenotype, making cancer cells more susceptible to treatment (85). The miR-23a∼27a∼24-2 cluster plays a critical role in promoting the inflammatory polarization of macrophages, particularly influencing the balance between pro-inflammatory (M1) and anti-inflammatory (M2) states in the context of ovarian cancer. This cluster, composed of miRs-23a, -27a, and -24-2, enhances M1 polarization, which is crucial for anti-tumor immune responses, while its deletion skews macrophages toward the immunosuppressive M2 state that supports tumor growth. In ovarian cancer models, the absence of this miRNA cluster in macrophages leads to increased tumor progression and poor outcomes, as the macrophages adopt an M2 phenotype, suppressing the immune response and aiding tumor evasion. Thus, the miR-23a cluster regulates macrophage plasticity, and targeting it could be a potential strategy to enhance tumor immunity by modulating macrophage polarization (86). Similarly, miR-141-3p accelerates OC progression by promoting cell proliferation, migration, and invasion while enhancing M2-like macrophage polarization by targeting the Keap1-Nrf2 pathway. MiR-141-3p is overexpressed in OC, leading to the downregulation of Keap1, a negative regulator of Nrf2. This, in turn, activates Nrf2, a transcription factor involved in antioxidant defense and tumor progression. The activation of Nrf2 facilitates the transformation of macrophages into the M2 phenotype, which supports tumor growth and metastasis. Inhibiting miR-141-3p disrupts this pathway, reducing M2 polarization and suppressing tumor development, making it a potential therapeutic target in OC (87). MiR-217 inhibits M2-like macrophage polarization in ovarian cancer by directly targeting and suppressing the secretion of IL-6, a key cytokine involved in promoting this immunosuppressive macrophage phenotype. In ovarian cancer tissues and cell lines, miR-217 is downregulated, which leads to increased IL-6 secretion and the activation of the JAK2/STAT3 signaling pathway, driving M2-like macrophage polarization. Overexpression of miR-217 reduces IL-6 levels, which in turn inhibits the JAK2/STAT3 pathway, decreasing M2 macrophage markers and promoting an immune environment less conducive to tumor progression. This mechanism highlights miR-217’s potential as a therapeutic target for disrupting the tumor-supportive microenvironment in ovarian cancer (88). In M1 macrophages (anti-tumor), LncRNA-Xist is upregulated, maintaining the M1 phenotype by inhibiting miR-101, which in turn allows the expression of C/EBPα and KLF6, two transcription factors that support M1 polarization. In contrast, miR-101 is highly expressed in M2 macrophages (pro-tumor) and suppresses C/EBPα and KLF6, promoting M1-to-M2 polarization. This shift enhances cancer cell proliferation and migration in ovarian cancer. Therefore, Xist acts as a competing endogenous RNA (ceRNA) by binding miR-101, preventing it from promoting the pro-tumor M2 phenotype, thereby reducing cancer progression (89). CircITGB6 contributes to cisplatin resistance in ovarian cancer by altering the tumor microenvironment, specifically by shifting TAMs toward the M2 phenotype, which is linked to tumor growth and immune suppression. Elevated levels of circITGB6 in cisplatin-resistant ovarian cancer cells form a complex with the RNA-binding protein IGF2BP2 and fibroblast growth factor 9 (FGF9) mRNA. This interaction stabilizes FGF9 mRNA, increasing its expression and secretion. Higher FGF9 levels promote the polarization of TAMs into the M2 phenotype, which supports tumor progression and limits the effectiveness of cisplatin by creating an immunosuppressive environment. This M2 macrophage-driven environment protects cancer cells from cisplatin-induced apoptosis, contributing to chemoresistance. Therapeutically, using antisense oligonucleotides to inhibit circITGB6 reduces M2 macrophage infiltration and enhances cisplatin sensitivity, positioning circITGB6 as a promising target to combat chemotherapy resistance in ovarian cancer (**Figure 4**) (90).

### Secreted tumor-derived factors inducing macrophage polarization

#### HE4

HE4 (human epididymis protein 4) is a glycoprotein commonly overexpressed in ovarian cancer, particularly in serous ovarian carcinomas, and is associated with aggressive tumor behavior and poor prognosis (91). Its overexpression creates a suppressive tumor immune microenvironment by promoting immune evasion and tumor progression. Specifically, reduces the infiltration and activation of cytotoxic T cells (CTLs) and natural killer (NK) cells, while enhancing the recruitment of M2 macrophages, which are immunosuppressive and promote tumor growth. Additionally, HE4 increases the expression of the immune checkpoint protein PD-L1 on both tumor cells and macrophages, through a novel posttranscriptional mechanism, further inhibiting immune responses against the tumor. This immune suppression driven by HE4 allows the cancer to evade immune detection, making it a potential target for therapeutic interventions, particularly in combination with PD-L1/PD-1 checkpoint inhibitors (**Figure 3**) (92).

#### Adrenomedullin

Adrenomedullin (ADM) plays a pivotal role in the polarization of macrophages toward the M2 phenotype in cancers, which contributes to tumor progression (93). In ovarian cancer, ADM is secreted by tumor cells and promotes the differentiation of macrophages into M2-like TAMs. These M2 macrophages, characterized by the expression of markers like CD206 and the secretion of anti-inflammatory cytokines such as IL-10 and CCL18, have pro-tumor functions. ADM-induced M2 macrophages support tumor growth, angiogenesis, and metastasis by enhancing the migration and invasion of ovarian cancer cells. The interaction between ADM and macrophages activates the RhoA signaling pathway in cancer cells, which leads to cytoskeletal rearrangements necessary for cell migration. Thus, ADM is a key factor linking macrophage polarization and ovarian cancer cell migration, highlighting its potential as a therapeutic target to inhibit TAM-mediated cancer progression (**Figure 4**) (94).

#### Periostin

Periostin (POSTN) is a secreted matricellular protein that is critical in promoting ovarian cancer metastasis (95). It enhances tumor progression by interacting with integrin receptors (specifically integrin β3 and β5) on cancer cells, which activates the ERK and NF-κB signaling pathways. This activation produces cytokines and chemokines that attract monocytes and promote their differentiation into tumor-associated M2 macrophages, known for their pro-tumor effects. POSTN also stimulates the expression of TGF-β2, which activates normal fibroblasts into cancer-associated fibroblasts (CAFs), further supporting tumor growth and invasion. These M2 macrophages and CAFs contribute to a tumor microenvironment that promotes immune evasion and metastasis, making POSTN a potential therapeutic target in ovarian cancer (**Figure 4**) (56).

#### TRAIL

TRAIL (TNF-related apoptosis-inducing ligand) is a member of the TNF superfamily known for its ability to induce cell death in transformed cells by binding to death receptors (DR4 and DR5) while sparing normal cells (96). TRAIL promotes the polarization of human macrophages toward a pro-inflammatory, tumor-fighting M1 phenotype by increasing the expression of M1 markers and reducing M2 markers. TRAIL enhances the cytotoxicity of macrophages against cancer cells through both DR4 and DR5 signaling pathways. Additionally, in cancer patients, high levels of TRAIL expression are associated with longer overall survival, particularly in cases with high tumor macrophage content, suggesting that TRAIL enhances anti-tumor responses by promoting M1 macrophage polarization in the tumor microenvironment (**Figure 4**) (97).

### Metabolic regulators influencing macrophage function

#### PGI2

Prostacyclin (PGI2) plays a pivotal role in the ovarian cancer microenvironment by mediating interactions between CAFs and TAMs, significantly influencing macrophage polarization and tumor progression. CAFs, the primary producers of PGI2 due to their high expression of prostacyclin synthase (PTGIS), release this bioactive lipid into the TME, where it binds to the PGI2 receptor (PTGIR) on TAMs, which express this receptor at high levels. Upon activation, PGI2 induces a mixed polarization state in TAMs, affecting both M1 and M2 macrophage phenotypes. Typically, M1 macrophages exhibit pro-inflammatory, anti-tumorigenic properties, while M2 macrophages are associated with immunosuppressive, pro-tumorigenic behavior. However, PGI2 signaling blurs these distinctions, as it promotes M1-like markers such as CD86 but represses pro-inflammatory M1 genes like TNF and FCGRs. Simultaneously, it enhances M2-like features, including the secretion of VEGF, a key factor in angiogenesis, while inhibiting the expression of other M2 markers like CD206 (MRC1). This polarization reduces the phagocytic capacity of TAMs and suppresses immune activation by downregulating cytokines critical for T cell and NK cell recruitment, such as CXCL10 and IL12A. Consequently, PGI2 skews TAMs toward a pro-tumorigenic, immunosuppressive phenotype that supports tumor growth, metastasis, and immune evasion. By reshaping the macrophage polarization landscape, PGI2 becomes a critical factor in ovarian cancer progression, and targeting its signaling pathway offers a potential therapeutic strategy to counteract tumor-promoting macrophage functions (**Figure 3**) (98).

#### Glutaminolysis

N-acetylaspartate (NAA) is a metabolite primarily known for its role in the brain but is also implicated in various cancers, including ovarian cancer (99). In glutaminolytic ovarian cancer cells, which are highly dependent on extracellular glutamine due to low glutamine synthetase (GS) levels, NAA is produced as a byproduct of altered metabolism. These cancer cells release NAA into the tumor microenvironment, where it acts in synergy with IL-10 to polarize TAMs toward an M2-like, protumoral state. NAA achieves this by inhibiting the NMDA receptor (NMDAR) on macrophages, leading to increased GS expression, which reinforces the M2-like phenotype. This metabolic crosstalk between cancer cells and macrophages supports tumor growth, immune evasion, and cancer progression (**Figure 4**) (100, 101).

#### mtDNA

Mitochondrial transcription factor B2 (TFB2M) is a key protein involved in the regulation of mitochondrial DNA (mtDNA) transcription and compaction. It functions as part of a mitochondrial transcription complex that includes mitochondrial transcription factors A (TFAM) and B1 (TFB1M), essential for mtDNA maintenance and the expression of genes critical for mitochondrial function (102). TFB2M specifically modulates both the transcription of mtDNA and the regulation of its copy number, playing a vital role in mitochondrial biogenesis and homeostasis. In ovarian cancer, overexpression of TFB2M has been linked to an immunosuppressive tumor microenvironment by promoting M2 macrophage infiltration. This process is mediated through cytosolic mtDNA stress, which occurs when excess mtDNA is released into the cytoplasm due to mitochondrial dysfunction or fission. The release of cytosolic mtDNA acts as a damage-associated molecular pattern (DAMP), activating the Toll-like receptor 9 (TLR9) pathway and leading to the activation of the NF-κB signaling pathway. This activation stimulates the secretion of IL-6, a pro-inflammatory cytokine known to promote the recruitment and polarization of macrophages into the M2 phenotype. M2 macrophages are associated with tumor progression due to their immunosuppressive and pro-tumorigenic activities, such as promoting angiogenesis, tissue remodeling, and suppression of cytotoxic immune responses (**Figure 3**) (73).

#### CYP24A1

CYP24A1, an enzyme crucial in vitamin D (VD) metabolism, significantly contributes to OC progression by influencing the polarization of TAMs. Increased CYP24A1 expression accelerates the breakdown of active vitamin D, limiting its role in supporting anti-tumor immune functions. This leads to a shift in TAMs from the pro-inflammatory, cancer-fighting M1 phenotype to the tumor-supporting M2 phenotype. M2 macrophages encourage tumor growth, angiogenesis, and metastasis, thereby driving OC progression. By altering vitamin D availability and TAM polarization, CYP24A1 promotes cancer advancement, making it a promising biomarker and therapeutic target in OC treatment (**Figure 3**) (103).

### Tumor suppressor-like factors with macrophage reprogramming potential

#### SORBS2

SORBS2 is an RNA-binding protein that plays a crucial role in suppressing the metastatic colonization of cancers by stabilizing tumor-suppressive transcripts (104). It binds to the 3’ untranslated regions (UTRs) of two key immunomodulatory molecules, WFDC1 and IL-17D, which are secreted factors that inhibit cancer metastasis. By stabilizing these transcripts, SORBS2 enhances their expression, thereby limiting the invasiveness of cancer cells and promoting a tumor-suppressive immune microenvironment. This action affects immune cell polarization, reducing the recruitment of tumor-promoting myeloid cells and M2-like macrophages, ultimately creating conditions unfavorable for metastasis. Thus, SORBS2 contributes to ovarian cancer suppression by linking tumor progression and immune regulation through its post-transcriptional stabilization of key immunomodulatory transcripts (**Figure 3**) (92).

#### PEDF

Pigment Epithelium-Derived Factor (PEDF) is a 50-kDa glycoprotein known for its potent anti-angiogenic and anti-tumor properties. In OC, PEDF levels are significantly reduced, and its low expression is associated with advanced disease and poor patient outcomes. PEDF delays ovarian cancer progression by modulating the immune microenvironment, specifically by influencing TAMs, which are typically polarized toward the M2 subtype that promotes tumor growth, angiogenesis, and immune suppression. PEDF overexpression shifts TAMs from the tumor-promoting M2 phenotype to the anti-tumor M1 phenotype, thereby inhibiting tumor growth and enhancing cancer cell apoptosis. This effect is mediated through the regulation of ATGL (Adipose Triglyceride Lipase) and the ERK1/2 signaling pathway. By reprogramming the macrophages in the tumor microenvironment, PEDF helps create an immune response more hostile to cancer cells, thus slowing ovarian cancer progression (**Figure 3**) (105).

### Extracellular and receptor-mediated regulators

#### Gelsolin

Gelsolin is an actin-binding protein involved in the regulation of the cytoskeleton, with two forms: intracellular and extracellular (plasma gelsolin, or pGSN) (106). In ovarian cancer, plasma gelsolin (pGSN) plays a significant role in conferring chemoresistance. pGSN is secreted by ovarian cancer cells and is often found in elevated levels in chemoresistant patients. It modulates the tumor microenvironment by interacting with immune cells, specifically TAMs. Normally, M1 macrophages have anti-tumor properties, producing nitric oxide (NO) and TNFα, which promote cancer cell death. However, pGSN interferes with this process by inducing apoptosis in M1 macrophages and reducing their production of NO and TNFα, thereby inhibiting their anti-tumor function. At the same time, pGSN does not affect M2 macrophages, which are pro-tumorigenic, creating an imbalance in the M1/M2 macrophage ratio. This shift toward a tumor-promoting environment helps cancer cells evade chemotherapy, increasing tumor survival, recurrence, and chemoresistance. As a result, pGSN serves as a key factor in reducing the effectiveness of chemotherapy in ovarian cancer patients (**Figure 4**) (107).

#### FPR2

Formyl peptide receptor 2 (FPR2) is a member of the G protein-coupled receptor (GPCR) family, involved in various physiological and pathological processes, including inflammation, immune responses, and cancer progression. In epithelial ovarian cancer (EOC), FPR2 significantly promotes cancer cell invasion, migration, and metastasis. It does so through activating the small GTPase RhoA, a key regulator of cytoskeletal dynamics and cellular movement. Overexpression of FPR2 in EOC cells enhances RhoA expression, leading to increased migratory capacity of the cancer cells. Additionally, FPR2 influences the tumor microenvironment by stimulating the secretion of Th2 cytokines, such as IL-4 and IL-10, which drive the polarization of macrophages into the M2 phenotype. M2 macrophages are associated with immune suppression, tumor progression, and angiogenesis, thereby facilitating EOC metastasis. In contrast, inhibiting RhoA reverses this process, promoting M1 macrophage polarization, which has anti-tumor properties. Thus, FPR2 promotes EOC progression by simultaneously enhancing cancer cell mobility through RhoA activation and fostering a pro-tumor immune environment via M2 macrophage polarization (108).

#### Copine 1

Copine 1 (CPNE1) is a calcium-dependent, membrane-binding protein that plays a critical role in promoting tumor progression in various cancers, including ovarian cancer (109). In ovarian cancer, CPNE1 is significantly upregulated and associated with poor prognosis. It regulates TAMs by promoting their polarization into the M2 phenotype, which is tumor-supportive. CPNE1 achieves this by upregulating markers such as CD163, CD206, and interleukin-10 (IL-10), which are characteristic of M2 macrophages. These M2 macrophages contribute to tumor growth and metastasis by creating a favorable tumor microenvironment, in contrast to M1 macrophages, which have anti-tumor properties. Thus, CPNE1’s role in macrophage polarization supports ovarian cancer progression and makes it a potential therapeutic target (**Figure 4**) (110).

## Inducing macrophage polarization into the M2 phenotype role of extracellular vesicles in macrophage polarization

Tumor-derived extracellular vesicles (EVs) significantly influence macrophage polarization in the tumor microenvironment. These EVs, released by cancer cells, transport bioactive components like miRNAs, proteins, and lipids that impact macrophage behavior. Tumor-derived EVs mainly drive macrophages to adopt the M2 phenotype, which is linked to immunosuppression, tissue repair, and the promotion of tumor growth. M2 macrophages contribute to tumor progression by supporting angiogenesis, enhancing metastasis, and dampening immune responses (111). On the other hand, M1 macrophages, which have pro-inflammatory and anti-tumor properties, are suppressed by these EVs. This capacity of tumor-derived EVs to push macrophages toward a tumor-supporting M2 state underlines their crucial role in shaping the tumor environment and advancing cancer development. By understanding these processes, new therapeutic strategies could focus on using EVs to reprogram macrophages into the M1 phenotype, potentially leading to anti-tumor outcomes. This phenomenon has been demonstrated across multiple ovarian cancer models, with findings from both *in vitro* and *in vivo* studies supporting the role of OV-EVs in immune modulation. However, the majority of these studies rely on preclinical models, and direct evidence from patient-derived EV samples remains limited, emphasizing the need for clinical validation (111). Ovarian cancer-derived extracellular vesicles (OV-EVs) promote cancer progression and angiogenesis primarily by inducing the polarization of macrophages into the M2 phenotype, which is associated with tumor growth and tissue repair. OV-EVs are taken up by macrophages, triggering their transformation into M2 macrophages. These M2 macrophages then secrete higher levels of vascular endothelial growth factor (VEGF), which binds to VEGF receptors (VEGFR) on endothelial cells, thereby stimulating angiogenesis, the formation of new blood vessels. This angiogenesis is crucial for supplying the growing tumor with nutrients and oxygen, further accelerating cancer progression. The OV-EVs also promote the infiltration of M2 macrophages into tumor tissues, enhancing their ability to support the tumor microenvironment, contributing to tumor metastasis, immune evasion, and overall disease progression (112). Cancer-associated fibroblasts (CAFs) are a key component of the tumor microenvironment and play a crucial role in cancer progression by influencing various biological processes. These fibroblasts, derived from normal stromal cells, undergo activation in the tumor context, where they secrete a variety of cytokines, growth factors, extracellular matrix, and EVs components that support tumor growth, invasion, and metastasis. CAFs can also modulate immune responses, particularly by interacting with TAMs (113). In ovarian cancer, they induce TAM polarization toward the M2-like phenotype, which is known for its immunosuppressive and pro-tumorigenic functions. This polarization is driven through the secretion of cytokines such as IL-33, IL-6, and TGF-β, which influence monocytes to differentiate into M2 macrophages. These M2 macrophages, in turn, secrete anti-inflammatory and tumor-promoting factors like IL-10 and TGF-β, further enhancing cancer cell invasion, metastasis, and immune evasion, thereby creating a tumor-promoting microenvironment (114).

### Proteins in EVs

**Figure 5:**
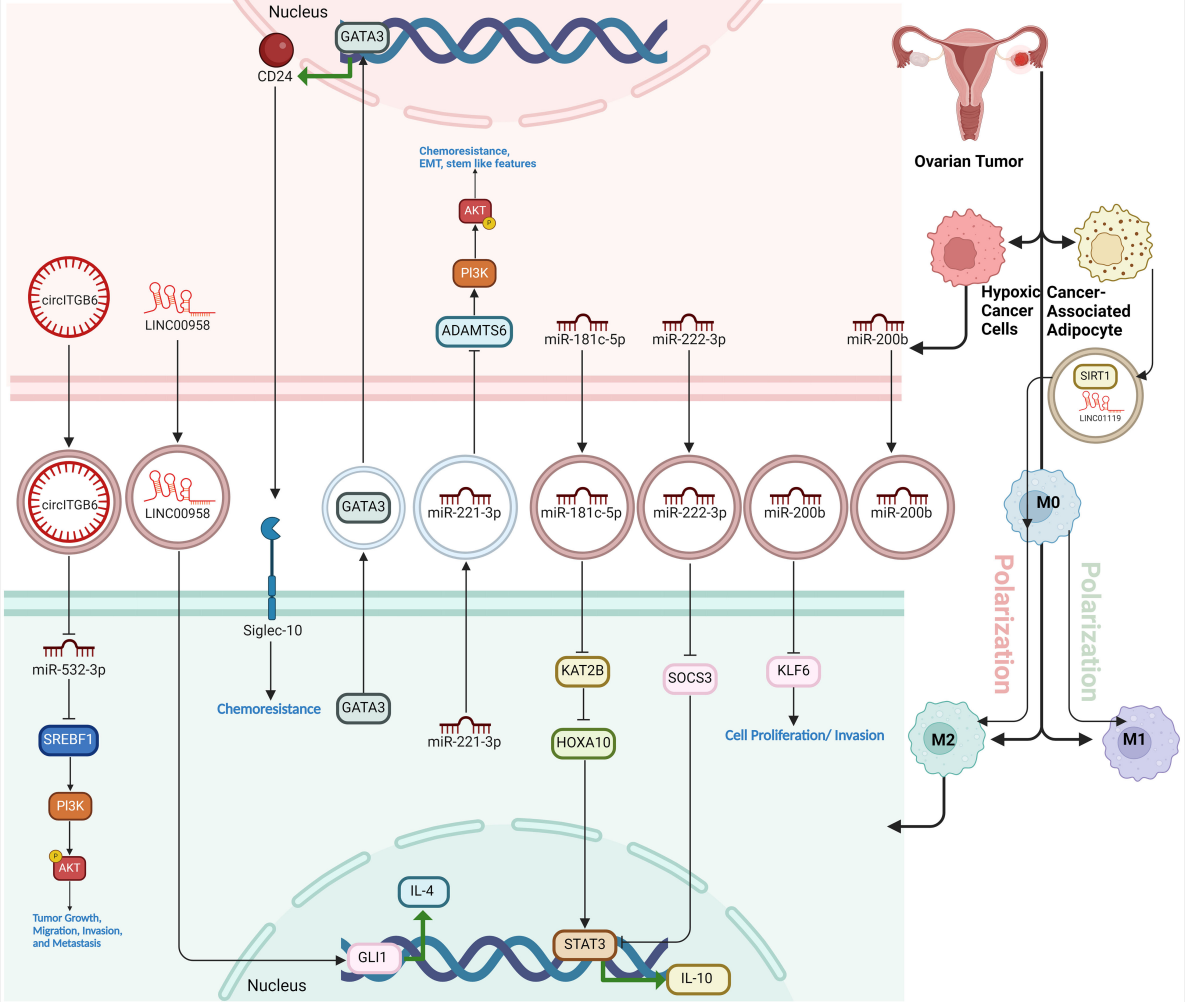
The role of EVs in macrophage polarization within the ovarian tumor microenvironment. Tumor-derived EVs from ovarian cancer cells and associated stromal cells (like cancer-associated adipocytes) carry various bioactive molecules, including miRNAs, circRNAs, and lncRNAs, which influence macrophage behavior. EV-encapsulated components drive macrophages toward an M2 immunosuppressive phenotype, promoting immune evasion, tumor progression, chemoresistance, and metastasis. Key pathways involved include the CD24/Siglec-10 axis and the PI3K/AKT signaling pathway, activated by transferred miRNAs and regulatory proteins, enhancing tumor-supportive functions of macrophages and contributing to a microenvironment conducive to cancer survival and invasion.

Sirtuin 1 (SIRT1) is a nicotinamide adenine dinucleotide (NAD+)-dependent deacetylase that plays a key role in regulating various cellular processes, including metabolism, aging, inflammation, and stress resistance (115, 116). In cancer, SIRT1 can influence tumorigenesis by modulating cell survival pathways and inhibiting tumor suppressor proteins. The delivery of SIRT1 by cancer-associated adipocyte-derived extracellular vesicles (CAA-EVs) significantly alters immune cell populations within the tumor microenvironment, contributing to ovarian cancer cell survival. Specifically, SIRT1 transferred via CAA-EVs promotes an increase in M2 macrophages, which are known for their immunosuppressive and tumor-promoting roles. These macrophages help create a favorable environment for tumor growth by dampening inflammatory responses and facilitating immune escape. Simultaneously, SIRT1-mediated activation of the CD24/Siglec-10 axis suppresses the activity of CD8+ T cells, crucial immune cells responsible for targeting and killing cancer cells. This dual modulation, enhancing M2 macrophages and reducing CD8+ T cell activity, creates an immune-suppressive environment that enables cancer cells to evade immune detection and survive, promoting tumor progression (117). GATA3 encapsulated within TAM-derived EVs, specifically from M2-polarized macrophages, promotes immune escape and chemotherapy resistance in OC cells by activating the CD24/Siglec-10 axis. M2 macrophages, known for their immunosuppressive and tumor-promoting roles, release EVs that transfer GATA3 to OC cells. Once inside the cancer cells, GATA3 acts as a transcription factor, upregulating CD24 expression. CD24 then interacts with Siglec-10, a receptor found on macrophages, particularly M2 TAMs, leading to the suppression of T cell-mediated immune responses. This interaction creates an immunosuppressive microenvironment, facilitating immune escape and allowing the tumor to evade immune detection. Moreover, the CD24/Siglec-10 axis enhances the tumor’s resistance to chemotherapy, particularly cisplatin, by reducing apoptosis in cancer cells (**Figure 5**) (118).

### MiRNAs in EVs

Extracellular vesicle-packaged miR-181c-5p from hypoxic EOC cells promote the M2 polarization TAMs through the KAT2B/HOXA10 axis. Specifically, miR-181c-5p is transferred to TAMs via EVs, where it targets and downregulates KAT2B, a key regulatory protein. This suppression of KAT2B increases the acetylation of HOXA10, which subsequently activates the JAK1/STAT3 signaling pathway. The activation of this pathway drives the M2 polarization of TAMs, which enhances tumor-promoting activities such as cell proliferation, migration, and invasion, contributing to the growth and metastasis of EOC (119). Likewise, miR-200b is significantly upregulated in plasma-derived exosomes of ovarian cancer patients and plays an oncogenic role by promoting macrophage M2 polarization. This polarization enhances the tumor-supportive environment, as M2 macrophages secrete anti-inflammatory cytokines that facilitate cancer cell proliferation and invasion. miR-200b achieves this by suppressing the expression of Kruppel-like factor 6 (KLF6), a tumor suppressor that normally inhibits M2 polarization. As a result, miR-200b indirectly promotes ovarian cancer progression by altering the immune landscape, making it a potential target for therapeutic intervention in ovarian cancer (18). The molecular mechanism of CD163+ TAM-derived exosome-induced cisplatin resistance in ovarian cancer ascites involves the transfer of exosomes containing miR-221-3p from TAMs to ovarian cancer cells. Upon uptake, miR-221-3p downregulates the expression of ADAMTS6, a tumor suppressor gene. This downregulation activates the AKT signaling pathway, which in turn promotes EMT, leading to the acquisition of cancer stem cell (CSC)-like characteristics and the upregulation of multidrug resistance (MDR) genes. EMT is characterized by increased levels of transcription factors such as SNAIL1 and ZEB1, as well as mesenchymal markers like Vimentin, while reducing the epithelial marker E-cadherin. This process enhances ovarian cancer cells’ resistance to cisplatin by promoting cell proliferation, migration, and survival, ultimately contributing to the poor prognosis in ovarian cancer. Overexpression of ADAMTS6 can inhibit this pathway, highlighting its potential as a therapeutic target for reversing drug resistance (19). Similarly, EOC-secreted exosomal miR-222-3p induces the polarization of macrophages into a TAM-like M2 phenotype, which supports tumor progression. Exosomes from EOC cells carry miR-222-3p, which is taken up by macrophages, leading to the downregulation of SOCS3, a negative regulator of the STAT3 signaling pathway. This suppression of SOCS3 activates STAT3, promoting M2 polarization characterized by increased expression of markers like CD206 and Arg-1, along with elevated IL-10 secretion and reduced IL-12 levels. These M2-like macrophages foster an immunosuppressive tumor microenvironment that enhances the proliferation, migration, and metastatic potential of ovarian cancer cells (120). Similarly, exosomes derived from hypoxic EOC cells are enriched with microRNA-940 (miR-940) and play a key role in inducing macrophage M2 polarization. Under hypoxic conditions, EOC cells increase the expression of miR-940, which is packaged into exosomes and delivered to nearby macrophages. Upon uptake, these exosomes drive macrophages to adopt an M2-like TAM phenotype, marked by higher expression of M2 markers such as CD163 and CD206. These M2-polarized macrophages, in turn, promote tumor progression by enhancing the proliferation and migration of EOC cells (**Figure 5**) (121).

### LncRNA in EVs

LINC01119, encapsulated by exosomes derived from cancer-associated adipocytes (CAA-Exo), plays a crucial role in promoting M2 polarization of macrophages, thereby facilitating immune escape in ovarian cancer. In a 3D co-culture cell-based model, mature adipocytes co-cultured with OC cells transform into CAAs, which release exosomes carrying LINC01119. These exosomes are taken up by macrophages, triggering their polarization into the immunosuppressive M2 phenotype. M2 macrophages, in turn, suppress the proliferation and cytotoxic activity of CD3+ T cells, enhance PD-L1 expression on OC cells, and reduce the immune system’s ability to target and destroy cancer cells. This process is mediated through the upregulation of SOCS5, a target of LINC01119, which further drives macrophage polarization and immune evasion, ultimately supporting tumor progression and immune resistance in ovarian cancer (122). Exosomal LINC00958 maintains OC cell stemness and induces M2 macrophage polarization by interacting with the Hedgehog signaling pathway through the GLI1 protein. LINC00958, a long non-coding RNA, is secreted by OC cells within exosomes and transferred to other cells in the tumor microenvironment. In OC cells, LINC00958 enhances stem cell-like properties by increasing the expression of stemness-associated markers, such as ALDH and NANOG, and promoting the formation of cancer stem cell spheres. This is mediated through its interaction with GLI1, a key transcription factor in the Hedgehog signaling pathway. LINC00958 binds to GLI1, promoting its nuclear translocation and activating the transcription of GLI1 target genes, such as SOX2, which are crucial for maintaining stemness in OC cells. Additionally, LINC00958-containing exosomes induce M2 macrophage polarization, a tumor-supportive immune phenotype characterized by markers like CD206 and the expression of immunosuppressive genes such as IL-10 and Arg-1. This polarization is also mediated via the Hedgehog/GLI1 pathway, where LINC00958 facilitates GLI1 activation, driving IL-4 transcription in macrophages and enhancing the M2 phenotype. Thus, exosomal LINC00958 plays a dual role in promoting OC cell stemness and reprogramming the immune environment to support tumor progression (**Figure 5**) (123).

### CircRNAs in EVs

Circ_C20orf11 enhances cisplatin (DDP) resistance in ovarian cancer by acting as a competing endogenous RNA that sponges miR-527, preventing it from suppressing YWHAZ, a protein associated with drug resistance and tumor progression. This inhibition allows YWHAZ to remain active, thereby promoting cancer cell survival and DDP resistance. Additionally, circ_C20orf11 is encapsulated in EVs derived from DDP-resistant ovarian cancer cells and transferred to macrophages in the tumor microenvironment. These EVs induce macrophage polarization toward the M2 phenotype, which creates a tumor-promoting and immunosuppressive environment, further contributing to chemoresistance. Silencing circ_C20orf11 reverses these effects, reducing DDP resistance, increasing apoptosis, and decreasing macrophage M2 polarization (124). EV-packaged circATP2B4 is critical in promoting EOC metastasis by mediating M2 macrophage polarization through the miR-532-3p/SREBF1 axis. EOC cells secrete EVs containing circATP2B4, which TAMs take up in the tumor microenvironment. Once inside the macrophages, circATP2B4 acts as a competing endogenous RNA (ceRNA) by binding to and inhibiting miR-532-3p, a microRNA that normally suppresses the expression of SREBF1, a key transcription factor involved in lipid metabolism and M2 polarization. By sponging miR-532-3p, circATP2B4 allows for the upregulation of SREBF1, which subsequently activates the PI3K/AKT signaling pathway. This signaling cascade promotes the M2 polarization of macrophages, transforming them into an immunosuppressive phenotype that supports tumor growth, migration, invasion, and metastasis. M2 macrophages secrete pro-tumorigenic cytokines and growth factors that foster an environment conducive to the EMT of EOC cells, enhancing their metastatic potential. Thus, circATP2B4 facilitates a feedback loop where EVs from EOC cells modulate the tumor microenvironment by reprogramming macrophages to an M2 phenotype, thereby promoting cancer progression and metastasis. This mechanism reveals circATP2B4 as a potential therapeutic target, as its inhibition could disrupt M2 polarization and hinder EOC metastasis (**Figure 5**) (125).

## Role of ovarian cancer-stem cells in macrophage polarization

Cancer stem cells (CSCs) play a critical role in shaping the tumor microenvironment by driving the polarization of macrophages toward the tumor-promoting M2 phenotype. CSCs achieve this by secreting chemokines, cytokines (such as CCL2, IL-6, IL-10, and TGF-β), and exosomes, which recruit macrophages and induce their differentiation into M2 TAMs (126). These M2 macrophages support tumor growth through immunosuppressive actions, angiogenesis, and extracellular matrix remodeling, while also secreting factors like IL-6 and TNF-α that reinforce CSC stemness and contribute to tumor progression and metastasis. Signaling pathways such as STAT3, TGF-β, and β-catenin/CCL2 are key mechanisms by which CSCs promote M2 polarization. This interaction creates a positive feedback loop where M2 macrophages, in turn, further enhance CSC survival, resistance to therapy, and tumor aggressiveness, making the CSC–TAM crosstalk a significant target for potential therapeutic strategies (127). OCSCs promote the polarization of macrophages into the tumor-promoting M2 phenotype through the secretion of cytokines rather than direct cell-to-cell contact. This process is primarily driven by the overexpression of COX-2 in OCSCs, which leads to an increased production of PGE2. The COX-2/PGE2 pathway activates JAK signaling pathway in macrophages, inducing their differentiation into the M2 phenotype. M2 macrophages, characterized by high levels of anti-inflammatory markers such as IL-10 and reduced pro-inflammatory factors like TNF-α, contribute to the immune suppression, tumor progression, and metastasis commonly seen in ovarian cancer. This mechanism reveals the role of OCSCs in shaping the tumor microenvironment, facilitating tumor growth and evasion of immune responses (128). OCSCs promote the M2 polarization of macrophages by modulating the PPARγ and NF-κB pathways. When OCSCs interact with macrophages, they increase the expression of M2 markers such as mannose receptor, IL-10, and arginase-1, while suppressing M1 markers like TNF-α, iNOS, and CD86. This shift is mediated by the activation of PPARγ, a nuclear receptor with anti-inflammatory properties, and the simultaneous inhibition of the pro-inflammatory transcription factor NF-κB. Blocking PPARγ with the antagonist GW9662 reverses this effect, reducing M2 marker expression and increasing M1 marker expression, indicating that PPARγ is crucial for OCSC-induced M2 polarization. This interaction helps create an immune-suppressive tumor microenvironment that favors cancer progression (51). OCSCs and macrophages engage in reciprocal interactions through the WNT signaling pathway within 3D engineered microenvironments, promoting pro-tumoral and malignant phenotypes. OCSCs drive the polarization of macrophages into an immunosuppressive M2-like phenotype, characterized by increased CD206 expression, via the secretion of WNT ligands and cytokines like IL-10. In turn, macrophages secrete WNT5B, which enhances the maintenance of the stemness properties of OCSCs, including elevated ALDH activity, chemoresistance, and invasiveness. This bidirectional WNT-mediated signaling forms a positive feedback loop, with CSC-derived WNT ligands promoting M2 macrophage activation and macrophage-derived WNT5B fostering the stemness and malignancy of OCSCs. This interaction not only supports tumor progression but also contributes to increased resistance to chemotherapy and a more invasive cancer phenotype, making the WNT pathway a potential therapeutic target in ovarian cancer (45). OCSLCs induce macrophage polarization toward the tumor-promoting M2 phenotype by altering the macrophages’ cytokine secretion profile and activating specific signaling pathways. When co-cultured with THP-1 macrophages, OCSLCs increase the expression of M2 markers such as CD163, while boosting the secretion of anti-inflammatory cytokines like IL-10 and pro-tumor factors such as VEGF and MMP-9, which are characteristic of M2 macrophages. At the same time, OCSLCs reduce the production of pro-inflammatory molecules like IL-12 and nitric oxide (NO), which are typical of the M1 macrophage phenotype. Central to this process is the secretion of interleukin-8 (IL-8) by OCSLCs, which activates the STAT3 signaling pathway in macrophages. This IL-8/STAT3 axis plays a crucial role in promoting the M2 polarization, fostering a tumor-friendly environment that supports cancer progression and stemness in ovarian cancer cells (61).

## Macrophage polarization and therapy resistance in ovarian cancer

Macrophage polarization plays a critical role in therapy resistance in cancer, particularly through the actions of TAMs within the tumor microenvironment. TAMs often adopt an M2-like (pro-tumor) phenotype, which promotes tumor progression and undermines the effectiveness of cancer therapies, including chemotherapy, immunotherapy, radiotherapy, and targeted therapy. M2-like TAMs secrete immunosuppressive cytokines, express inhibitory ligands like PD-L1, and protect tumor cells from drug-induced cell death, thereby contributing to multidrug resistance. Reprogramming these macrophages to an M1-like (anti-tumor) phenotype is considered a promising strategy to enhance therapeutic responses and overcome cancer drug resistance (129).

### Chemotherapy resistance

Chemotherapy induces polarization of TAMs in ovarian cancer, shifting them from an immunosuppressive, tumor-promoting state to a more pro-inflammatory, antitumor phenotype. This polarization is characterized by a reduction in alternatively activated (M2-like) macrophages, which are typically associated with poor prognosis, and an increase in classically activated (M1-like) macrophages that support adaptive immune responses (130). After neoadjuvant chemotherapy (NACT), TAMs showed increased expression of pro-inflammatory pathways, including activation of the inflammasome and secretion of cytokines like IL-1β, which can promote T-cell activation and enhance the overall immune response against the tumor. These chemotherapy-induced changes in TAM polarization suggest that TAMs can aid adaptive immunity by fostering a more favorable tumor microenvironment, potentially enhancing the effectiveness of immunotherapies and improving clinical outcomes in ovarian cancer (28). However, it should be noted that the platinum sensitivity of ovarian cancer cells does not influence their ability to induce M2-type macrophage polarization. Both platinum-sensitive (A2780) and platinum-resistant (A2780Cis and A2780Dox) ovarian cancer cell lines were found to polarize macrophages toward an M2-like phenotype, regardless of their sensitivity to cisplatin. M2-type macrophages, which are typically associated with tumor progression and an immunosuppressive environment, were consistently induced by the cancer cells in coculture systems. This suggests that the ability of ovarian cancer cells to shape the tumor microenvironment by promoting M2-type macrophages is independent of their platinum resistance status, pointing to a robust mechanism by which ovarian cancer cells modulate the immune response to support tumor growth (131). Macrophages stimulated by paclitaxel-resistant ovarian cancer cells (OCTR) adopt an M2 phenotype, which is associated with tumor-promoting activities. These macrophages express higher levels of M2 markers, such as mannose receptor (MR) and triggering receptor expressed on myeloid cells 2 (Trem2), compared to macrophages stimulated by non-resistant ovarian cancer cells. The conditioned medium from these M2-polarized macrophages further increases the resistance of ovarian cancer cells to paclitaxel, contributing to a feedback loop where both the cancer cells and macrophages enhance each other’s survival and resistance mechanisms. This suggests that M2-polarized TAMs play a significant role in reinforcing chemoresistance in ovarian cancer (35).

### Immunotherapy resistance

Targeting the macrophage population to reprogram or enhance TAMs into an M1-polarized, CXCL10-secreting state offers a promising new therapeutic approach, particularly for ovarian cancer subtypes like clear cell carcinoma (CCC), which are often marked by immune exclusion and poor prognosis. M1-polarized TAMs have potent antitumor properties and play a key role in recruiting and activating T-cells through the production of chemokines like CXCL10. This recruitment strengthens the immune response against tumors. However, CCC typically lacks these beneficial M1 macrophages, contributing to its resistance to treatment and reduced immune cell infiltration. By reprogramming the macrophages already present in CCC tumors to shift toward an M1 phenotype, this immune-deficient environment could be transformed into one that supports a more robust immune response. This could potentially be achieved through therapies that stimulate M1 polarization, such as IFNγ or specific cytokine modulators, while simultaneously inhibiting M2 macrophage activity, which tends to promote tumor growth. This strategy could enhance the efficacy of immunotherapies by increasing tumor immunogenicity, thereby improving responsiveness to treatments such as immune checkpoint inhibitors and macrophage-targeted therapies, including CSF1R and TREM2 inhibitors. This approach offers new therapeutic prospects for challenging OC subtypes like CCC (68). IL-4 plays a crucial role in establishing an immunosuppressive TME by promoting macrophage polarization toward an M2-like phenotype, which contributes to ICB resistance in OvCa. Despite its relatively low expression in tumors, IL-4 signaling can have profound effects on TME programming, as even a small fraction of IL-4-producing cancer cells can drive significant immune suppression. Recent studies utilizing CRISPR screens and Perturb-map analysis have identified IL-4 as a key mediator of immune resistance, particularly in its ability to sustain an immunosuppressive niche that limits effector T cell activity. Notably, IL-4R blockade has shown promise in reversing these effects. While IL-4R inhibition has primarily been explored in inflammatory conditions, its potential application in cancer therapy is gaining attention. The clinically approved IL-4Rα antagonist, dupilumab, has demonstrated efficacy in modulating immune responses and is currently under clinical investigation in combination with PD-1 blockade for lung cancer. Preclinical data suggest that dual blockade of IL-4R and PD-1 leads to a more permissive immune landscape, characterized by reduced macrophage-mediated immune suppression and enhanced T cell infiltration. Given the established role of IL-4 in driving immune evasion in OvCa, IL-4R blockade may represent a promising strategy to overcome resistance to immunotherapy, particularly when combined with anti-PD-1 therapy. Future clinical trials evaluating IL-4R inhibitors in OvCa could provide critical insights into their efficacy in reversing macrophage-mediated immunosuppression and enhancing response rates to immune checkpoint inhibitors (72).

## Macrophage polarization-related biomarkers in ovarian cancer

The traditional M1/M2 paradigm suggests that M1 macrophages, induced by IFN-γ, TNF-α, and TLR signaling, promote anti-tumor immunity through the production of pro-inflammatory cytokines and reactive oxygen species. In contrast, M2 macrophages, driven by IL-4, IL-10, and TGF-β, are associated with tumor progression via immunosuppression, angiogenesis, and tissue remodeling. However, TAMs within the tumor microenvironment (TME) frequently exhibit features of both M1 and M2 macrophages, demonstrating phenotypic plasticity that enables them to adapt dynamically to their microenvironment (132). This mixed polarization reflects the complexity of signaling networks and the intricate interplay of cytokines, chemokines, growth factors, and metabolites in the TME (133). As a result, the M1/M2 classification fails to fully capture the range of functional states exhibited by TAMs in ovarian cancer (134).

Recent single-cell RNA sequencing (scRNA-seq) studies have further challenged this binary M1/M2 classification by revealing diverse TAM subpopulations with distinct transcriptional profiles and functional properties (135). These studies demonstrate that TAMs may simultaneously exhibit immunosuppressive characteristics while producing pro-inflammatory mediators, emphasizing their ability to perform multiple, often contradictory roles within the TME (136). For example, some TAMs interact with regulatory T cells and tumor cells to establish an immunosuppressive TME, reinforcing the notion that macrophages do not neatly fit into M1 or M2 categories (133). This transcriptional heterogeneity underscores the necessity of moving beyond the simple M1/M2 framework toward more nuanced approaches that capture the true complexity of TAM biology. In ovarian cancer ascites, TAMs frequently display mixed-polarization phenotypes, co-expressing markers associated with both M1 and M2 macrophages (132). For instance, CD163, a marker commonly associated with M2 macrophages, correlates with ascitic levels of IL-6 and IL-10, cytokines that can mediate both pro- and anti-inflammatory responses (134). Interestingly, transcriptional profiling of TAMs indicates that CD163 expression does not always correlate with other M2 markers, and many TAMs also express M1-associated genes, further exposing the limitations of the classical M1/M2 classification. scRNA-seq analyses have identified unique TAM subpopulations with distinct transcriptional programs, revealing macrophage states that do not conform to the traditional dichotomy and that exhibit immune evasion properties (136). Given this complexity, it is imperative to redefine macrophage classification in the context of ovarian cancer. Our review will emphasize the importance of characterizing TAM phenotypes beyond the M1/M2 framework by leveraging advanced technologies such as scRNA-seq, spatial transcriptomics, and functional assays. These integrative approaches will enable a more precise understanding of TAM biology and uncover novel therapeutic strategies targeting specific TAM subpopulations, ultimately improving treatment outcomes for ovarian cancer patients. By incorporating these cutting-edge methodologies, we aim to provide a more accurate and nuanced perspective on macrophage polarization in ovarian cancer, thereby enhancing the rigor and impact of our research.

Macrophage polarization markers offer significant benefits in cancer prognosis by helping to identify the functional states of TAMs, which play key roles in tumor biology. M1 macrophages, with tumoricidal properties, support immune responses and tumor destruction, while M2 macrophages promote tumor growth, survival, and immune evasion. By identifying these subtypes through markers such as CD68 (general macrophage marker), CD163, CD204, and CD206 (M2 markers), researchers can assess the tumor microenvironment and predict patient outcomes. In general, M2 markers are associated with worse overall survival in various cancers. These markers also help in understanding the tumor’s anatomical location, invasion behavior, and the impact of macrophage presence on treatment outcomes, enabling more personalized treatment strategies (137). In this section, we have summarized the macrophage polarization-related biomarkers in ovarian cancer.

### CD163

CD163 is a surface receptor primarily found on cells of the monocytic lineage, particularly macrophages, and is traditionally associated with the M2 (alternatively activated) polarization of macrophages (138). In ovarian cancer, CD163 plays a significant role in the tumor microenvironment, where it is upregulated on TAMs found in malignant ascites. While CD163 is typically regarded as a marker of M2 polarization, which is linked to anti-inflammatory and tissue repair functions, its role in ovarian cancer is more complex. The study by Reinartz et al. shows that despite high CD163 expression, TAMs in ovarian cancer exhibit a mixed-polarization phenotype that includes features of both M1 (pro-inflammatory) and M2 macrophages. CD163 expression is also correlated with elevated levels of the cytokines IL-6 and IL-10 in ascites, both of which induce CD163 expression and are associated with poorer clinical outcomes, such as early relapse. Thus, in ovarian cancer, CD163 is not strictly indicative of M2 polarization but is a marker of a more nuanced macrophage phenotype linked to disease progression and immune modulation (139).

### LILRB1

LILRB1 (Leukocyte Immunoglobulin-Like Receptor Subfamily B1) is an inhibitory receptor involved in immune regulation by interacting with MHC class I molecules, particularly HLA-G (140). It is predominantly expressed on immune cells such as dendritic cells, macrophages, monocytes, NK cells, and certain T cell subsets, where it plays a role in suppressing immune activation and maintaining immune tolerance, often contributing to immune suppression (141). In OC, LILRB1 is primarily expressed on immune cells (ICs) rather than tumor cells (TCs) and is strongly associated with an immunosuppressive tumor microenvironment. This environment is characterized by increased infiltration of M2 macrophages, reduced dendritic cell activation, and impaired function of CD8+ T cells, which are essential for anti-tumor responses. High infiltration of LILRB1+ ICs correlates with poorer clinical outcomes, including more advanced disease stages, shorter progression-free and overall survival, and diminished responses to both chemotherapy and immune checkpoint inhibitors (ICIs) like anti-PD-1/PD-L1 therapies. Moreover, patients with higher LILRB1+ IC levels tend to show resistance to platinum-based chemotherapy. These findings suggest that LILRB1+ ICs act as independent prognostic indicators and may predict weaker responses to immunotherapy, positioning LILRB1 as a promising target for future therapeutic approaches in OC treatment (142)

### MUC2

MUC2 is a mucin glycoprotein that is aberrantly overexpressed in ovarian cancer, contributing to tumor progression by influencing immune cell behavior, particularly macrophages (143). In ovarian cancer, high MUC2 expression is associated with the polarization of TAMs toward the M2 phenotype, which supports tumor growth, metastasis, and immune suppression, in contrast to the M1 phenotype, which has anti-tumor effects. MUC2 interacts with TAMs, promoting the expression of COX-2 in these cells, leading to increased production of PGE2. PGE2 further polarizes macrophages toward the M2 type, creating a feedback loop that exacerbates tumor progression and is linked to poorer patient outcomes. Thus, MUC2 plays a critical role in altering the immune landscape within the tumor microenvironment, favoring cancer growth (144).

### Semaphorin4D

Semaphorin4D (SEMA4D) is a protein that plays a significant role in the tumor microenvironment of epithelial ovarian cancer (EOC). Overexpression of SEMA4D is associated with poor prognosis, as it correlates with advanced disease stages, chemotherapy resistance, and reduced overall and progression-free survival. In the context of EOC, SEMA4D influences the differentiation of monocytes into M2 macrophages, which are known for their pro-tumor activity. M2 macrophages promote tumor progression by fostering an environment that supports cancer growth, metastasis, and angiogenesis. Studies have shown a strong association between high levels of SEMA4D and increased counts of M2 macrophages in both primary tumors and ascites, further linking SEMA4D to the aggressive behavior of EOC and suggesting its potential as a target for prognostic and therapeutic strategies (145).

### FGF18

FGF18 (Fibroblast Growth Factor 18) has been identified as an important prognostic and therapeutic biomarker in high-grade serous ovarian cancer (146). Amplification of the chromosomal region 5q31-5q35.3, where FGF18 is located, is strongly associated with poor prognosis in ovarian cancer patients. FGF18 overexpression promotes tumor progression by enhancing tumor cell migration, invasion, and tumorigenicity. It modulates the tumor microenvironment by activating NF-κB signaling, which leads to the production of oncogenic cytokines and chemokines that enhance angiogenesis and recruit TAMs, particularly M2-polarized macrophages known for their tumor-supportive roles. This contributes to a more aggressive tumor phenotype. In patient samples, FGF18 overexpression correlates with increased microvessel density and TAM infiltration, confirming its involvement in driving tumor progression. Thus, FGF18 is a potential therapeutic target, and its inhibition could offer a novel treatment approach for ovarian cancer (146).

### RBMS3

RBMS3, a RNA-binding protein with tumor-suppressing functions, shows reduced expression in epithelial ovarian cancer (EOC), and this decrease is linked to poorer outcomes for patients, such as shorter overall survival and progression-free survival (147). Research indicates that when RBMS3 is overexpressed, it can slow tumor growth, reduce the ability of cancer cells to spread, and promote cell death. Additionally, RBMS3 affects the immune environment of tumors by decreasing the presence of certain immune-suppressing cells, such as regulatory T cells, myeloid-derived suppressor cells, and M2 macrophages, which are often associated with supporting tumor growth. In contrast, it increases M1 macrophages, which aid the body’s immune response against tumors. These findings suggest that RBMS3 has a role in controlling tumor progression and shaping the immune environment, making it a potential target for new cancer therapies (148).

### FMO2

TRPM2 is a non-selective cationic channel that belongs to the transient receptor potential (TRP) ion channel family, known to play a role in various physiological and pathological processes, including tumor progression. In OC, TRPM2 is significantly overexpressed and is associated with poor prognosis. It is linked to immune regulation within the tumor microenvironment, particularly influencing the polarization of macrophages. TRPM2 expression is positively correlated with M2 macrophages, which are tumor-promoting and linked to immunosuppression, rather than M1 macrophages, which have tumor-suppressing functions. This suggests that TRPM2 may promote tumor progression by fostering an immunosuppressive microenvironment through M2 macrophage polarization, making it a potential target for immunotherapy in OC (149).

### ALOX5AP

ALOX5AP (Arachidonate 5-Lipoxygenase Activating Protein) is an enzyme involved in the production of leukotrienes, which are immune-modulating lipid mediators. In serous ovarian cancer (SOC), ALOX5AP is highly expressed and is associated with poor prognosis and aggressive disease features, such as lymphatic invasion, resistance to platinum chemotherapy, and poor survival rates. ALOX5AP promotes tumor progression by influencing the tumor microenvironment, specifically by driving M2 macrophage polarization, which supports immunosuppression and tumor growth. Elevated ALOX5AP levels are strongly correlated with the presence of M2 macrophages, which are known to promote immune evasion, metastasis, and chemoresistance in ovarian cancer. Targeting ALOX5AP may reprogram the immune microenvironment, offering a potential therapeutic approach by combining ALOX5AP inhibitors with immunotherapy to counteract M2 macrophage-mediated immunosuppression and improve patient outcomes (150).

### Interleukin-6

Interleukin-6 (IL-6) is a pro-inflammatory cytokine that plays a key role in immune responses, inflammation, and hematopoiesis (151). In the context of advanced ovarian cancer, IL-6 is heavily involved in the tumor microenvironment, where it contributes to cancer-related anemia (CRA). CRA is a form of anemia commonly seen in cancer patients, driven by inflammation and characterized by low serum iron levels and high ferritin, often resulting from the dysregulation of iron metabolism. Macrophages, which are immune cells that can polarize into two main phenotypes, M1 (pro-inflammatory) or M2 (anti-inflammatory), are central to this process. In ovarian cancer, M1 TAMs predominate and are stimulated by IL-6 to release hepcidin, a hormone that regulates iron homeostasis by restricting iron availability, which worsens anemia. This polarization of macrophages toward the M1 phenotype in the tumor microenvironment amplifies inflammation and contributes to CRA by promoting iron sequestration and reducing hemoglobin levels. Targeting IL-6 to modulate macrophage polarization and reduce hepcidin production may offer a therapeutic strategy to manage CRA in patients with advanced ovarian cancer (152).

## Targeting macrophage polarization in ovarian cancer

**Figure 6:**
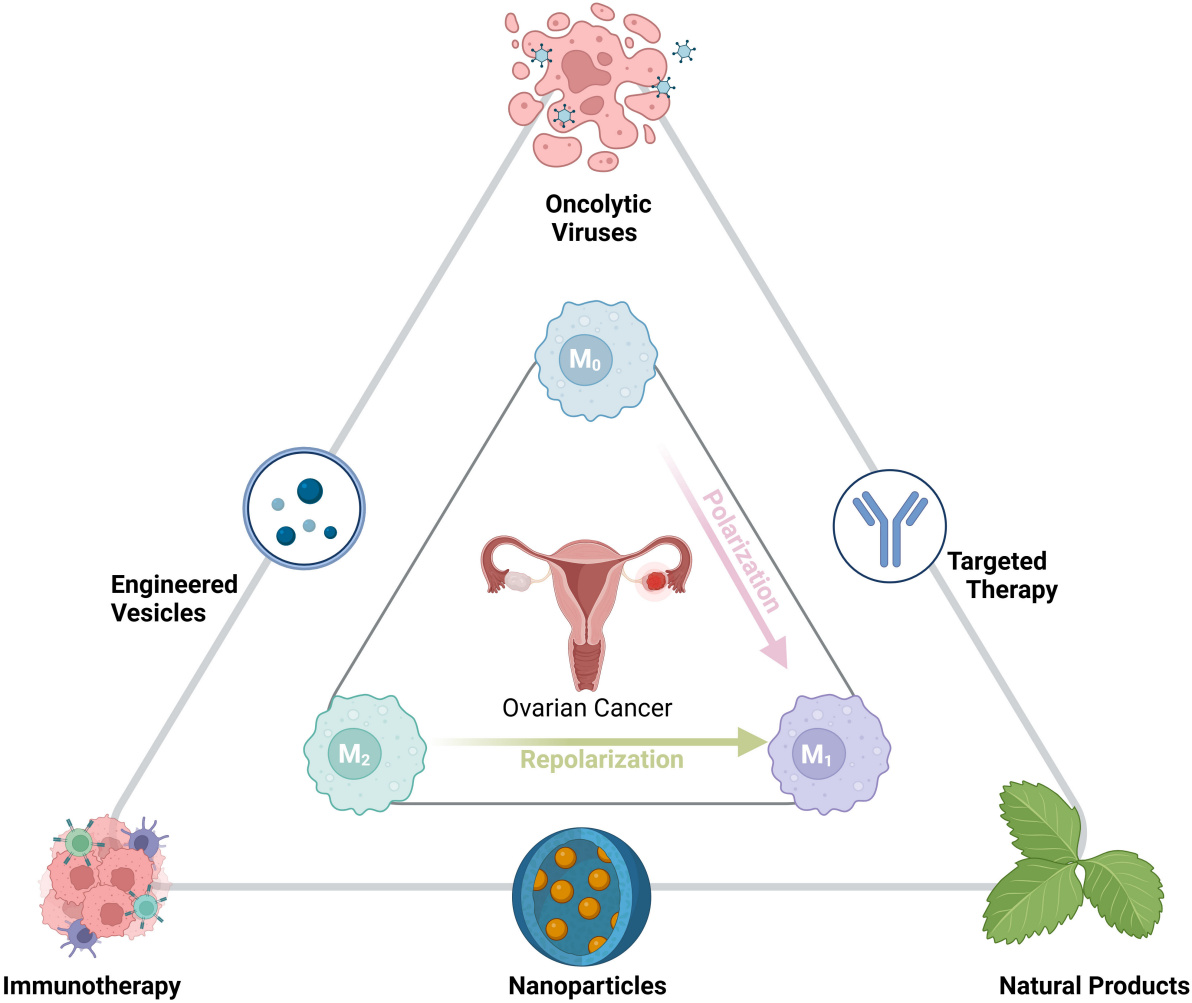
Therapeutic strategies targeting ovarian cancer macrophage polarization: Potential interventions, including oncolytic viruses, engineered vesicles, immunotherapy, nanoparticles, targeted therapy, and natural products. These approaches aim to modulate macrophage polarization, shifting from pro-tumorigenic M2 to anti-tumorigenic M1 states, enhancing the immune response against ovarian cancer.

Targeting macrophage polarization is promising in cancer therapy because macrophages, especially TAMs, play a dual role in the tumor microenvironment. While M1 macrophages exhibit antitumor properties by promoting immune responses and killing cancer cells, M2 macrophages generally support tumor growth by fostering angiogenesis, suppressing immune responses, and aiding metastasis. Since TAMs are often skewed toward the M2 phenotype in established tumors, reprogramming or repolarizing them from the tumor-promoting M2 type to the tumor-suppressing M1 type can inhibit tumor progression and enhance the efficacy of immunotherapies. This approach harnesses the natural plasticity of macrophages to shift them toward an antitumoral role, offering a novel and potent strategy for improving cancer treatment outcomes (153). In this section, we review the therapeutic potential of targeting macrophage polarization in ovarian cancer (**Table 1**) (**Figure 6**):

**Table 1:** Treatment targeting macrophage polarization in ovarian cancer.

Treatment name	Drug type	Cell Lines	Model (*in vivo*/*in vitro*)	Effects on Macrophage Polarization	Highlights	Ref.
oncoVV-AVL	Oncolytic vaccinia virus harboring *Aphrocallistes vastus* lectin	A2780, SKOV3	*In vitro* (cell lines), *in vivo* (tumor-bearing nude mice)	Promotes M1 macrophage polarization through increased IFN-γ levels	- Induces apoptosis and autophagy in ovarian cancer cells - Increases ROS production, promoting viral replication - Elevates serum IFN-γ levels, enhancing the immune response through M1 macrophage polarization - Suppressed by NADPH, which reduces ROS, IFN-γ, and M1 polarization	(205)
Oncolytic Adenovirus (OV)	Oncolytic virus	ID8 mouse ovarian cancer cells	*In vivo* (mouse model with malignant ascites)	Promotes M1 macrophage polarization and reduces M2 macrophages	- OV reduced ascites formation and prolonged survival in mice - Enhanced T cell infiltration and activation (especially CD8+ T cells) - Reprogrammed the immune microenvironment toward proinflammatory status - Combination therapy (OV + anti-PD-1 + PLX3397) further improved T cell activity and delayed ascites progression	(206)
Human Macrophage-Engineered Vesicles (MEVs)	Vesicles derived from M1-polarized macrophages	Caov-3, OVCAR3, SKOV3 (ovarian cancer), RAW264.7 (murine macrophage)	*In vitro* (cell co-cultures), *in vivo* (mouse ovarian cancer xenografts)	Converts M2 macrophages to M1-like pro-inflammatory macrophages	- MEVs repolarize M2 macrophages to M1 state, reducing ovarian cancer cell viability - MEVs target ovarian tumors in mouse xenografts - Potential for drug delivery and immune modulation	(200)
Exosome-Cisplatin (M1/M2)	Chemotherapy-loaded Exosome	A2780, A2780/DDP	*In vitro*	M1-exosomes promote tumoricidal M1 polarization.	Cisplatin-loaded M1-exosomes increase cytotoxicity, reducing IC50 by 1.4-2 fold in resistant cells.	(201)
CpG-ODNs + Anti-PD-1 Antibody	TLR9 Agonist + Immune Checkpoint Inhibitor	IGROV-1 (human ovarian cancer), RAW264.7 (macrophage cell line)	*In vivo* (xenograft ovarian cancer model in athymic nude mice) and *In vitro*	Drives macrophage polarization toward an immunoregulatory (M2b-like) phenotype, increasing expression of CD206, IL-10, and other immunosuppressive markers.	Combination of CpG-ODNs and anti-PD-1 antibodies reduces antitumor efficacy, as macrophages acquire a suppressive phenotype, impairing immune response.	(156)
Murlentamab	Low-fucosylated anti-AMHRII monoclonal antibody	SKOV3-R2+, COV434-R2+ (ovarian cancer cells)	*In vivo* (humanized mouse model) and *in vitro*	Promotes reprogramming of TAMs toward an M1-like phenotype with increased expression of M1 markers (CD80, TLR2) and reduced M2 markers (CD163, CD206, CD36).	Murlentamab shifts TAMs to an anti-tumor M1-like phenotype, enhancing ADCC and activating both innate and adaptive immune responses, including T cell activation and reduced Treg levels.	(157)
Murlentamab + Pembrolizumab	Combination of anti-AMHRII monoclonal antibody + anti-PD-1 antibody	SKOV3-R2+, COV434-R2+ (ovarian cancer cells)	*In vivo* (humanized mouse model) and *in vitro*	Further enhances the M1-like polarization of TAMs and amplifies the activation of CD8+ T cells and Th1 CD4+ T cells, along with reduced tumor growth.	The combination of murlentamab with pembrolizumab potentiates macrophage reprogramming, increasing CD8+ T cell activation and reinforcing anti-tumor immune responses *in vitro* and *in vivo*.	
PPAB001	Bispecific Antibody	BT-474, SK-OV-3	*In vivo* (mouse xenografts)	Increases M1/M2 ratio; promotes shift from M2 (tumor-promoting) to M1 (anti-tumor) macrophages	Potent synergy by blocking CD47/SIRPα and CD24/Siglec-10 pathways, enhances macrophage phagocytosis and anti-tumor immune responses; reduces tumor growth in ovarian cancer models.	(159)
CAR-T targeting FRβ+ TAMs	Chimeric Antigen Receptor (CAR)-T Cells	ID8 (ovarian cancer), B16 (melanoma), MC38 (colon adenocarcinoma)	*In vivo* (mouse models), Ex vivo (patient samples)	Promotes M1-like macrophages by depleting immunosuppressive M2-like FRβ+ TAMs	Reprograms TME to enhance pro-inflammatory monocytes and CD8+ T cell response; enhances effectiveness of tumor-targeted CAR-T therapy when used sequentially.	(160)
IκBα-MnNPs (mannose-decorated nanoparticles)	siRNA-loaded nanoparticles targeting IκBα	ID8 (C57Bl/6), TBR5 (FVB), NGL-BMDMs	*In vivo* (mouse models of ovarian cancer); *in vitro* (macrophage cultures)	Shifted macrophages from M2 (pro-tumor) to M1 (anti-tumor) phenotype by activating NF-κB pathway	- Biodistribution: MnNPs were preferentially delivered to TAMs in tumors and ascites, with minimal off-target delivery to the spleen or other organs. - Late-stage model (ID8): After a 3-day treatment, there was no significant reduction in tumor weight, but decreased ascites volume and altered immune cell composition were observed, indicating a therapeutic effect. - Aggressive model (TBR5): Biweekly treatment significantly reduced ascites accumulation and slightly decreased tumor burden, with increased M1 markers and inflammatory cytokines (CXCL9, TNF-α). - Immune Infiltration: Enhanced infiltration of CD8+ T cells into tumors, suggesting potential synergy with T cell-based therapies. - Safety: No significant toxicity was observed with repeated MnNP treatments (AST, ALT, and BUN within normal ranges).	(171)
miR497/TP-HENPs	Hybrid nanoparticle codelivery of TP (Triptolide) and miR497	SKOV3, SKOV3-CDDP (cisplatin-resistant)	Both *in vivo* (mouse model) and *in vitro*	Induces M2 to M1 polarization, promoting anti-tumor immune response	Efficient delivery system for overcoming chemoresistance; targets PI3K/AKT/mTOR pathway, increases ROS, depletes GSH, and minimizes systemic toxicity.	(172)
IRF5/IKKβ mRNA Nanoparticles	Targeted mRNA nanocarrier	Murine bone marrow-derived macrophages, THP-1 (human monocytes)	*In vivo* (ovarian cancer, melanoma, glioblastoma models in mice) and *in vitro*	Reprograms M2-like TAMs to M1 phenotype, leading to anti-tumor immune response and reduced tumor burden	Targeted mRNA delivery system induces M1 polarization in TAMs without systemic toxicity, improves immune response, and shows safety for repeated dosing	(173)
VSSP	Nanoparticle (ganglioside NAcGM3 + Neisseria meningitidis OMV)	Mouse ovarian cancer cells (Luc+MOSEC); Human TAMs from EOC ascites	*In vivo* (murine ID8 syngeneic model), *In vitro* (human TAMs and healthy donor neutrophils)	Induced M1-like polarization; reduced M2 markers (CD206), increased M1 markers (iNOS2, CD86); stimulated TNF-α and IL-1β secretion	VSSP treatment modulated TAM and granulocyte populations in TME, decreased suppressor phenotype of myeloid cells, partially reversed T cell suppression, and showed potential for combined immunotherapy.	(174)
Vorinostat	HDAC inhibitor	TOV-21G, TOV-112D (EAOC cell lines)	Both *in vitro* (cell lines) and *in vivo* (EAOC mouse model)	Decreased M2 macrophage polarization via reduced IL-10 levels	Vorinostat effectively inhibits HDAC6 expression, induces cancer cell apoptosis, and reduces EAOC tumor size by blocking the ARID1A6488delG/HDAC6/IL-10 pathway.	(177)
Plinabulin	Microtubule-targeting agent	MC38 (murine), EMT6 (murine), HuT 78 (human)	*In vivo* (murine), *in vitro* (human and murine)	Promotes M1-like polarization; increases CD80, CD86, IL-1β, IL-6, and IL-12; reduces IL-10 and IL-4	Plinabulin enhances anti-tumor immunity by promoting M1-like macrophages, activating JNK pathway, and directly killing tumor cells in a Fas/Fas-L dependent manner; effective even in T cell-deficient environments.	(178)
Erastin	Ferroptosis inducer	SKOV3, HO-8910	*In vitro* (cell culture) and *in vivo* (mouse model)	Promotes M2 polarization of TAMs through STAT3 activation, increasing IL-8 secretion	- Erastin enhances metastatic potential in ferroptosis-resistant OC cells via M2 polarization of macrophages. - The STAT3/IL-8 signaling axis is key in promoting OC cell migration and invasion. - IL-8 blockage reduces erastin-induced metastasis, suggesting STAT3/IL-8 as a potential therapeutic target.	(179)
Paclitaxel	Antineoplastic agent	B16 (Melanoma), 4T1 (Breast cancer), RAW 264.7, primary BMDMs	*In vitro* (cell culture) and *in vivo* (mouse models of breast and melanoma tumors)	Promotes M1 macrophage polarization through TLR4 signaling, suppressing M2 markers and enhancing M1 markers like TNFα and IL12	- Paclitaxel reprograms M2-polarized TAMs to an M1-like antitumor phenotype via TLR4. - Shows antitumor activity by altering TAM profiles and enhancing immune response. - Suggests potential for combination with immunotherapies for improved clinical outcomes.	(180)
AZD5153	BRD4 Inhibitor	CT26, THP-1, RAW264.7, ID8	*In vitro* (cell culture) and *in vivo* (mouse models with CT26 and ID8 ovarian tumors)	Shifts TAMs from M2 (pro-tumor) to M1 (pro-inflammatory) phenotype by inhibiting MAF transcription factor via BRD4, increasing IL-12 and decreasing IL-10 levels	- AZD5153 enhances antitumor immunity by repolarizing TAMs to an M1-like phenotype. - Downregulates PD-L1 on TAMs, sensitizing ovarian cancer cells to anti-PD-L1 therapy. - Promotes activation of CD8+ cytotoxic T cells, offering a potential combination strategy with immune checkpoint inhibitors for treating ovarian cancer.	(181)
Digitoxin	Cardiac Glycoside	SKOV3, HUVECs	*In vitro* (cell culture with macrophage-conditioned media)	Did not affect M1/M2 macrophage polarization but inhibited cancer and endothelial cell migration	- Inhibits migration and tube formation of endothelial cells (HUVECs) and ovarian cancer cells (SKOV3) without altering macrophage polarization. - Shows anti-angiogenic properties by inhibiting FAK phosphorylation in cancer and endothelial cells. - Potential for repositioning as an anticancer drug in invasive cancers due to selective effects on cancer and TME.	(182)
BP1003	Antisense Oligodeoxynucleotide (ASO)	SK-OV-3 (ovarian cancer), BT549 (breast cancer), SK-BR-3 (breast cancer)	*In vitro*, *In vivo* (PDX models)	Inhibits polarization of monocytes into M2 macrophages; no effect on M1 macrophages	BP1003 reduces STAT3 expression, enhances sensitivity to paclitaxel and 5-FU, reduces tumor growth, and blocks pro-tumorigenic M2 macrophage polarization, with potential in immunotherapy.	(183)
Cardamonin	Natural chalcone	SKOV3, A2780	*In vitro* & *in vivo* (mice)	Decreases M2 polarization by inhibiting mTOR/STAT3	Cardamonin reduced pro-tumor activity of TAMs, decreased M2 markers (CD163, CD206), and suppressed IL-6, VEGFα, MMP2, MMP9 secretion.	(186)
Verbascoside (VB)	Phenylpropanoid glycoside	SKOV3, A2780	*In vitro* & *in vivo* (mouse xenograft)	Promotes M1 polarization via CCN1-AKT/NF-κB pathway	VB suppresses OC cell proliferation and migration, induces apoptosis, and promotes M1 macrophage polarization by inhibiting CCN1 and the AKT/NF-κB pathway.	(193)
Triptolide (TPL)	Natural compound (Chinese herb)	A2780/DDP	*In vitro* & *in vivo* (mouse xenograft)	Inhibits M2 polarization via PI3K/AKT/NF-κB pathway	TPL reduces migration and invasion of drug-resistant OC cells by inhibiting M2 TAM polarization, particularly via PI3K/AKT/NF-κB pathway. Shows synergistic effect with DDP.	(189)
Kaempferia parviflora (KP)	Plant Extract	TOV-21G, THP-1	*In vitro*	Inhibits TNF-α-induced cytokine release (MCP-1, IL-6)	KP extract inhibits NF-κB nuclear translocation, upregulates IκB, and reduces cytokine production in TOV-21G cells. Also reduces phosphorylation of ERK1/2 and AKT, leading to decreased monocyte (THP-1) migration, reducing TAM infiltration.	(192)
Astragaloside IV	Natural Extract	THP-1, SKOV3	*In vitro*	Suppresses IL-4/IL-13-induced M2 polarization of macrophages	Inhibits HMGB1-TLR4 signaling in M2 macrophages, antagonizing their pro-tumor functions. Reduces M2 markers (e.g., TGF-β, MMP-9) and ovarian cancer cell proliferation, migration, and invasion facilitated by M2 macrophages.	(194)
Neferine	Natural compound from *Nelumbo nucifera*	HUVEC, RAW264.7, THP-1	*In vitro* (cell culture), *In vivo* (xenograft and CAM models)	Inhibits M2 macrophage polarization by decreasing CD206 expression, reducing the release of pro-angiogenic factors	Promotes autophagy in endothelial cells via mTOR/p70S6K inhibition, reduces tumor vascularization, and inhibits angiogenesis and M2-macrophage polarization, improving survival indicators in HGSOC models	(190)
Deoxyschizandrin	Natural lignan	A2780, SKOV3, OVCAR3, THP-1	*In vitro* (cell culture)	Inhibits M2 polarization, reducing CD163 and CD209 expression	Induces G0/G1 cell cycle arrest in ovarian cancer cells by suppressing cyclin E, and increases ROS levels, inhibiting Akt pathway. Suppresses pro-tumor factors (MMP-9, RANTES, VEGF) in TAMs, limiting tumor-supportive macrophage activity.	(191)
9-Hydroxycanthin-6-one	β-carboline alkaloid	A2780, SKOV3, OVCAR3	*In vitro*	Inhibits M2 macrophage polarization; decreases MCP-1, RANTES	Induces apoptosis in ovarian cancer cells through caspase- and ROS-dependent pathways; reduces recruitment of TAMs by inhibiting chemokines MCP-1 and RANTES; suppresses M2 polarization of TAMs and reduces cancer-promoting factors like MMP-2, MMP-9, and VEGF.	(197)
Onionin A (ONA)	Natural compound from onions	SKOV3, ES2, RMG1, murine ovarian cancer model	*In vitro* and *in vivo*	Inhibits M2 macrophage polarization and STAT3 activation	Suppresses ovarian cancer cell proliferation and macrophage-induced protumor effects by inhibiting STAT3; reduces IL-10 and other M2 markers in macrophages; enhances sensitivity to chemotherapy drugs (e.g., CDDP, PTX) in cancer cells.	(188)
Corosolic Acid (CA)	STAT3 Inhibitor	SKOV3, RMG-1, ES-2	*In vitro*	Inhibits M2 polarization of macrophages	Enhances chemosensitivity in ovarian cancer cells by inhibiting STAT3 signaling; reduces tumor-promoting macrophage interactions with cancer cells; synergistic with chemotherapy agents like paclitaxel and cisplatin.	(187)
Polyunsaturated Fatty Acids (PUFAs)	Lipid-derived Compound	OVKATE, OVSAHO, ES-2, A2780cp, ID8, THP-1, U937	*In vivo*, *in vitro*	Promotes M2-like TAM polarization via inhibition of RhoA-YAP1 signaling pathway	PUFAs from ovarian cancer ascites enhance M2-like TAM polarization by inhibiting RhoA-YAP1 signaling, leading to tumor progression. The restoration of YAP1 expression through MST1/2 inhibition with XMU MP1 promotes M1-like polarization, increases CD8+ T cell infiltration, and reduces metastasis.	(195)
SeviL	β-galactoside-binding lectin	RAW264.7 (macrophage), THP-1 (human monocyte), RBL-1 (rat basophil), HeLa (ovarian cancer)	*in vitro*	Induces M1 polarization in RAW264.7 macrophages, promotes pro-inflammatory cytokine release	SeviL binds to GA1 glycans on macrophages, triggering JAK/STAT and MAPK pathways. This leads to increased IL-6, TNF-α, and other pro-inflammatory cytokines and chemokines, as well as morphological changes (elongation) in macrophages. SeviL also has dose-dependent effects: it promotes proliferation at low doses (3-25 μg/mL) and cytotoxicity at higher doses (>25 μg/mL).	(207)
Solanum lyratum Thunb (SLT) Ethanol Extract	Natural Product (Traditional Chinese Medicine)	A2780, SKOV3 (ovarian cancer cells); M0 macrophages (THP-1 derived)	*In vitro*	SLT induces M1 polarization of M0 macrophages and inhibits M2 polarization. CD86+ (M1 marker) cells increase, while CD206+ (M2 marker) cells decrease, enhancing pro-inflammatory macrophages that suppress tumor growth.	SLT reduces ovarian cancer cell viability through ROS-mediated p53 activation, causing G1 cell cycle arrest, apoptosis, and inhibition of EMT. SLT’s anticancer mechanism is likely mediated by ROS accumulation, which activates the p53 pathway, leading to cell cycle arrest, apoptosis, and reduced migration/invasion. Furthermore, SLT modulates the tumor immune microenvironment by promoting M1 macrophage plarization and suppressing M2 polarization. These effects may aid in discovering new anti-ovarian cancer therapies.	(196)

### Immunotherapy

Shaping the polarization of TAMs is emerging as a promising approach in cancer immunotherapy due to TAMs’ critical role in the TME. TAMs predominantly exhibit an M2-like phenotype, which promotes tumor progression by supporting angiogenesis, immune suppression, and metastasis (154). By reprogramming these M2-like TAMs into the pro-inflammatory, tumor-inhibiting M1 phenotype, researchers aim to enhance anti-tumor immune responses and overcome resistance to therapies such as immune checkpoint blockade (ICB) and CAR-T cell therapy. Targeting specific signaling pathways, such as JAK/STAT, PI3Kγ/AKT, and NF-κB, which regulate TAM polarization, along with innovative strategies like nanocarriers, exosomes, and CAR-macrophage therapy, could significantly improve the efficacy of cancer treatments, particularly in solid tumors where immunosuppression is a major challenge (155). In an ovarian cancer model, the combination of TLR9 agonists (CpG oligodeoxynucleotides, CpG-ODNs) and anti-PD-1 antibodies can influence macrophage polarization by shifting them toward an immunoregulatory phenotype. TLR9 agonists activate innate immune responses by engaging toll-like receptors on macrophages, stimulating them to express pro-inflammatory molecules. However, when combined with anti-PD-1 antibodies, macrophages interact with the antibody’s Fc domain through their Fc receptors, which leads to their reprogramming. This interaction drives the macrophages toward an M2b-like phenotype, characterized by increased expression of immunosuppressive markers such as CD206 and IL-10. These macrophages, instead of promoting antitumor immunity, suppress the immune response, reducing the efficacy of CpG-ODN treatment. Thus, the combination of TLR9 stimulation and PD-1 blockade reshapes macrophages into a more suppressive, tumor-promoting state, limiting the potential therapeutic benefit of this regimen in ovarian cancer (156). Murlentamab, a low-fucosylated anti-Müllerian hormone type II receptor (AMHRII) antibody, exhibits potent anti-tumor activity in ovarian cancer by reprogramming TAMs and activating T cells. The low-fucosylation of murlentamab enhances its ability to bind to Fc receptors on immune cells, which promotes antibody-dependent cell-mediated cytotoxicity (ADCC) and phagocytosis (ADCP). When murlentamab opsonizes AMHRII-expressing ovarian tumor cells, it shifts TAMs from an immunosuppressive, tumor-promoting M2 phenotype to a pro-inflammatory, anti-tumor M1-like phenotype. This reprogramming results in increased expression of M1 markers (CD80, TLR2) and the production of pro-inflammatory cytokines and chemokines, such as IL-12 and IFNγ, while reducing M2 markers (CD163, CD206). Additionally, murlentamab enhances the recruitment and activation of adaptive immune cells, specifically increasing the activation of CD8+ T cells and promoting a Th1-biased CD4+ T cell response, while reducing regulatory T cells (Tregs). This dual activation of the innate and adaptive immune systems contributes to its robust anti-tumor effects in ovarian cancer (157). Anti-CRD4-MR scFv #G11 is a human recombinant single-chain variable fragment (scFv) antibody that specifically targets the carbohydrate recognition domain 4 (CRD4) of the Mannose Receptor (MR, CD206) on macrophages. In ovarian cancer, tumor-released mesothelin, which is linked to a glycosylphosphatidylinositol (GPI) anchor, binds to MR on macrophages, driving their polarization toward the TAM phenotype, which supports tumor growth and suppresses the immune response. By blocking this interaction, anti-CRD4-MR scFv #G11 effectively inhibits the polarization of macrophages into TAMs and preserves their pro-inflammatory, anti-tumor M1-like characteristics. This reprogramming of macrophages enhances their capacity to fight the tumor, promoting a more favorable immune response against ovarian cancer (158).

PPAB001 is a bispecific antibody fusion protein designed to simultaneously target and block CD47 and CD24, two critical immune checkpoints that inhibit macrophage-mediated phagocytosis of cancer cells. Both CD47 and CD24 are overexpressed in various cancers, including ovarian cancer, where they deliver “don’t eat me” signals to macrophages, preventing the immune system from effectively eliminating tumor cells. PPAB001 disrupts the interaction between CD47 and its receptor SIRPα, as well as between CD24 and Siglec-10, thereby promoting the phagocytic activity of macrophages against cancer cells. In ovarian cancer models, PPAB001 has shown enhanced efficacy compared to therapies that block only CD47 or CD24 individually. It not only increases the proportion of tumor-infiltrating macrophages but also shifts the balance from tumor-promoting M2 macrophages to tumor-fighting M1 macrophages. This change is accompanied by an upregulation of pro-inflammatory cytokines such as interleukin-6 (IL-6) and tumor necrosis factor-alpha (TNF-α), further enhancing the anti-tumor immune response. The dual blockade by PPAB001 effectively inhibits tumor growth in both mouse models and human xenograft models of ovarian cancer, suggesting its potential as a powerful immunotherapy approach (159). CAR-T cells engineered to specifically target and deplete FRβ+ TAMs in ovarian cancer effectively reprogram the TME by eliminating the immunosuppressive M2-like macrophage subset. The depletion of these FRβ+ TAMs shifts the balance toward a more pro-inflammatory state by promoting the infiltration and activation of M1-like macrophages, which are known for their antitumor properties. This reprogramming of the TME fosters a terrain that supports enhanced antitumor immunity, particularly by increasing the recruitment and activation of endogenous tumor-specific CD8+ T cells. These T cells exhibit greater infiltration into the tumor, higher expression of pro-inflammatory cytokines like IFN-γ and TNF-α, and improved tumor control. By converting the TME into a more immune-supportive environment, this approach not only suppresses tumor progression but also augments the effectiveness of other immunotherapies, enhancing overall antitumor immunity (160).

CSF1R, a receptor tyrosine kinase, plays a critical role in the differentiation and maintenance of TAMs, particularly the M2-polarized subtype, which contributes to immune suppression and tumor progression. TAMs harboring activated CSF1R release tumorigenic cytokines, creating an immunosuppressive microenvironment that hinders antitumor immunity. Pharmacological inhibition of CSF1R has therefore emerged as a promising strategy to modulate TAM function and enhance immunotherapy efficacy. Several preclinical and clinical studies have demonstrated that CSF1R inhibitors, including small-molecule tyrosine kinase inhibitors (e.g., PLX3397, GW2580, and Ki20227) and monoclonal antibodies targeting CSF1R or its ligand CSF1, can effectively deplete or reprogram TAMs, thereby improving tumor immune contexture and enhancing the efficacy of ICIs (161).

A previous study reported that CSF1 blockade in ovarian cancer models resulted in a significant reduction in CD163+ M2-like TAMs, with a concomitant increase in M1-polarized macrophages producing CXCL10, a chemokine crucial for T-cell recruitment. This shift in the TAM phenotype was associated with improved antitumor responses and enhanced T-cell infiltration. Furthermore, in high-grade serous ovarian cancer (HGSC), high CSF1 mRNA levels correlate with a dense infiltration of CD163+ TAMs, as evidenced by Nanostring gene expression analysis and immunohistochemical staining. This suggests that CSF1R blockade may be particularly beneficial for HGSC patients with high CSF1 expression (68). Clinical studies have also highlighted the therapeutic potential of CSF1R inhibitors in various cancers, including ovarian cancer. The FDA-approved CSF1R inhibitor PLX3397 (pexidartinib) has demonstrated clinical efficacy in reducing TAM-mediated immunosuppression, and early-phase trials have evaluated its combination with ICIs to further enhance immune responses (162). Additionally, GW2580, a selective CSF1R inhibitor, has been shown to normalize tumor vasculature and reduce malignant ascites in experimental ovarian cancer models by limiting M2 macrophage-mediated vascular permeability. Notably, this treatment increased the proportion of M1 macrophages expressing IL12 and IFNγ, leading to a more immunostimulatory tumor microenvironment (36). Beyond CSF1R inhibition, targeting TREM2, another macrophage-associated immunoregulatory receptor, has also emerged as a promising approach. TREM2 is expressed on CSF1R+ TAMs and modulated by CSF1, suggesting that dual inhibition of CSF1R and TREM2 may further potentiate macrophage repolarization and tumor immune surveillance. Preclinical studies have demonstrated that TREM2 blockade enhances the efficacy of ICIs, delays tumor progression, and promotes a favorable immune landscape in ovarian cancer (68).

Given the intratumoral heterogeneity of TAM polarization in ovarian cancer, a strategic combination of macrophage-targeted therapies, such as CSF1R and TREM2 inhibitors, with immunotherapies holds promise for improving clinical outcomes. By reprogramming TAMs toward an M1 phenotype, these interventions may help overcome immune exclusion, particularly in subtypes like clear cell carcinoma (CCC), where CXCL10-producing macrophages are scarce. Thus, leveraging CSF1R blockade in conjunction with ICIs or cytokine-based therapies represents a compelling avenue for enhancing immunotherapy efficacy in ovarian cancer.

### Single-cell RNA sequencing insights

Recent advancements in single-cell RNA sequencing (scRNA-seq) have significantly expanded our understanding of the complex and heterogeneous landscape of tumor-associated macrophages (TAMs) within the ovarian tumor microenvironment (TME). While our review extensively explores the molecular mechanisms and therapeutic potential of targeting macrophage polarization in ovarian cancer, the inclusion of recent scRNA-seq-based characterizations of TAM subpopulations provides critical insights into how distinct macrophage subsets contribute to tumor progression, immune evasion, and therapy resistance.

Several key scRNA-seq studies have delineated diverse TAM populations in ovarian cancer, revealing novel macrophage states and their functional implications. It has been demonstrated that ovarian tumor macrophages exhibit transcriptional diversity associated with immune evasion mechanisms, particularly through interactions with regulatory T cells and tumor cells to establish an immunosuppressive TME. This aligns with the predominance of M2-like macrophages in ovarian cancer, which foster immune suppression via IL-10, TGF-β, and VEGF signaling. These findings also reinforce the importance of identifying macrophage subtypes that exhibit differential responses to immunotherapy, suggesting that certain subsets may resist reprogramming efforts aimed at converting M2 macrophages into a pro-inflammatory M1 phenotype (163, 164). In a complementary study, researcher utilized scRNA-seq to classify macrophage populations in ovarian cancer based on their metabolic and immunoregulatory signatures. Their findings revealed that a subset of TAMs expresses high levels of oxidative stress response genes and metabolic regulators, which could contribute to their persistence in the TME and their role in therapy resistance. Our reviewer highlights similar trends, particularly in the discussion of PTEN-deficient tumors, where macrophage polarization is influenced by metabolic shifts, further reinforcing the idea that metabolism-targeted therapies could play a role in modulating TAM activity (165).

Vazquez-Garcia et al. and Hornberg et al. provided additional insights into the ontogeny of TAMs, distinguishing between monocyte-derived and tissue-resident macrophage subsets within ovarian tumors. Their findings suggest that tissue-resident macrophages may exhibit unique pro-tumor functions, distinct from infiltrating monocytes that differentiate into macrophages within the tumor. These studies provide a foundation for refining macrophage-targeting therapies by considering the origin and functional plasticity of macrophage populations. This is particularly relevant to discussion on macrophage recruitment pathways, such as CCL2-CCR2 and CXCL12-CXCR4, which influence monocyte migration into tumors and could be leveraged to alter the macrophage composition within the TME (166, 167).

Emerging spatial transcriptomics approaches have further refined our ability to map macrophage interactions within the ovarian tumor niche. The presence of distinct macrophage subtypes in different anatomical locations within tumors suggests that their functional roles may be context-dependent. The significance of the Wnt/β-catenin pathway and its role in macrophage-mediated immune suppression, and recent scRNA-seq studies suggest that these pathways may be differentially active in macrophage populations residing in peritoneal metastases compared to those in primary tumors. Moreover, focusing on immune exclusion in ovarian cancer, where tumors exhibit a low infiltration of cytotoxic T cells, is supported by findings from these scRNA-seq studies, which have identified macrophage populations that act as physical and biochemical barriers to T-cell infiltration. The data presented in Yeh et al. and Zheng et al. further emphasize the role of TAMs in modulating immune checkpoint activity, particularly through PD-L1 upregulation. This provides additional rationale for combining TAM-targeting therapies with immune checkpoint inhibitors to enhance the immune response against ovarian cancer (163, 165).

The incorporation of these recent scRNA-seq findings into our understanding of macrophage polarization in ovarian cancer underscores the complexity and adaptability of TAMs within the TME. While our review emphasizes key molecular regulators of macrophage polarization and potential therapeutic targets, these new studies highlight the need for a refined, subset-specific approach to TAM-targeted therapies. The diversity of macrophage states observed in ovarian cancer suggests that therapies aiming to reprogram TAMs should consider both their metabolic states and tissue residency status. As spatial and functional mapping of macrophages continues to evolve, integrating these insights into clinical strategies will be critical for enhancing the efficacy of existing and emerging immunotherapies.

### Nanoparticles

Nanotechnology offers significant advancements in OC therapy by enabling more targeted, effective, and less toxic treatments. Nanocarriers such as liposomes, dendrimers, polymer nanoparticles, and micelles can deliver chemotherapeutic agents directly to cancer cells, minimizing damage to healthy tissue and improving drug bioavailability (168). Functionalized nanoparticles can specifically target OC cells, enhancing the efficacy of treatments like paclitaxel and cisplatin while reducing side effects. Additionally, nanosensors improve early detection of OC biomarkers, enabling timely diagnosis. Nanoparticles are also used in imaging techniques to enhance tumor localization, and gene therapy approaches, such as siRNA delivery, help overcome drug resistance. Overall, nanotechnology enhances the precision and effectiveness of OC treatment, making it a promising tool in cancer therapy (169, 170). Mannose-decorated nanoparticles (MnNPs) are a specialized drug delivery system designed to target TAMs in the tumor microenvironment, particularly in ovarian cancer. These nanoparticles are coated with mannose, a sugar molecule that binds to the CD206 mannose receptor, which is overexpressed on immunosuppressive M2 macrophages. By delivering therapeutic agents like siRNA that targets IκBα, MnNPs can effectively trigger the NF-κB signaling pathway in these macrophages, repolarizing them from a tumor-promoting M2 phenotype to a tumor-fighting M1 phenotype. This repolarization increases inflammatory responses, promoting anti-tumor immunity and enhancing the body’s ability to fight cancer. In cancer therapy, MnNPs offer a targeted approach, reducing the immunosuppressive nature of the tumor microenvironment, improving the effectiveness of immune-based therapies, and potentially decreasing tumor growth and ascites accumulation with minimal off-target effects (171). A recent study developed a bioinspired hybrid nanoparticle system, miR497/TP-HENPs, combining exosomes and liposomes to co-deliver the chemotherapy agent triptolide (TP) and microRNA-497 (miR497) for treating cisplatin-resistant OC. The hybrid nanoparticles target tumors effectively, overcoming common drug resistance mechanisms by inhibiting the PI3K/AKT/mTOR pathway, generating ROS, and depleting glutathione (GSH), leading to enhanced cancer cell apoptosis. Additionally, miR497/TP-HENPs promote macrophage polarization toward an anti-tumor M1 phenotype, supporting immune-mediated tumor suppression. *In vivo*, the nanoparticles exhibited efficient tumor targeting and minimized systemic toxicity, suggesting a promising approach to treating chemoresistant OC and potentially other resistant cancers (172). Targeted mRNA nanocarriers encoding IRF5 and IKKβ can effectively reprogram TAMs from an immunosuppressive M2 phenotype to a pro-inflammatory M1 phenotype, thereby promoting an anti-tumor immune response. Using biodegradable polymeric nanoparticles (NPs) engineered for TAM targeting via CD206 mannose receptor binding, researchers delivered *in vitro*-transcribed mRNA encoding IRF5, a transcription factor crucial for M1 polarization, and its activating kinase, IKKβ. In murine models of ovarian cancer, melanoma, and glioblastoma, the IRF5/IKKβ NPs localized within tumor sites, where they reprogrammed TAMs, reduced M2 macrophage presence, and increased inflammatory cytokine production. This polarization enhanced tumoricidal activity while minimizing systemic toxicity, as demonstrated by the absence of significant inflammatory side effects, making the treatment safe for repeated administration. Importantly, this approach effectively inhibited tumor progression, prolonged survival in treated mice, and showed potential as an adjunctive therapy in combination with other immunotherapies (173). Lastly, VSSP (Very Small Size Particles) is a nanoparticle-based immunomodulator composed of the ganglioside NAcGM3 and outer membrane vesicles derived from Neisseria meningitidis. It acts as a Toll-like receptor (TLR-2/TLR-4) agonist and has been studied for its ability to modulate immune responses within the TME. In the context of murine ovarian tumors, VSSP abrogates immune suppression driven by tumor-associated myeloid cells by reducing the accumulation of suppressive TAMs and granulocytic cells, both of which hinder effective T cell-mediated anti-tumor immunity. VSSP promotes the polarization of TAMs from an immunosuppressive M2-like state to a pro-inflammatory M1-like state, which is associated with enhanced anti-tumor immune activity. This M1 polarization boosts the production of inflammatory cytokines and reduces the TAMs’ ability to suppress CD8+ T cell responses, making the immune system more effective in combating the tumor. In addition, VSSP stimulates peritoneal inflammation by increasing the presence of inflammatory monocytes and granulocytes, further aiding the anti-tumor response. In human studies, VSSP similarly induces M1 polarization in TAMs from ovarian cancer patients, enhancing immune activation and reducing the suppressive nature of the tumor microenvironment (174).

### Targeted therapies

In recent years, targeted therapies for ovarian cancer have advanced significantly, focusing on precision delivery to reduce toxicity and overcome drug resistance. Key developments include antibody-drug conjugates (ADCs), which bind to specific antigens on cancer cells to deliver cytotoxic drugs, as well as folate, peptide, and aptamer-drug conjugates that exploit ovarian cancer’s folate receptor overexpression for targeted delivery. These approaches have shown potential to increase therapeutic efficacy, reduce side effects, and target advanced, metastatic, and resistant ovarian cancers, though challenges like tumor environment and changing antigen expression require further refinement for clinical success (175, 176). Vorinostat is a histone deacetylase inhibitor (HDACi) that suppresses HDAC6 expression, thereby disrupting the ARID1A6488delG/HDAC6/IL-10 signaling pathway implicated in endometriosis-associated ovarian carcinoma (EAOC). In EAOC, the ARID1A6488delG mutation enhances HDAC6 expression, which leads to increased secretion of the anti-inflammatory cytokine IL-10, facilitating M2 macrophage polarization. M2 macrophages create an immunosuppressive environment that supports tumor growth and progression. By inhibiting HDAC6, Vorinostat reduces IL-10 levels, which decreases M2 macrophage polarization and consequently limits tumor growth. This pathway-specific effect positions Vorinostat as a promising therapeutic approach for targeting immune modulation in EAOC (177). Likewise, plinabulin is a novel microtubule-targeting agent that binds to the colchicine site of β-tubulin, destabilizing microtubules in a unique way to influence immune cells in the tumor environment. In ovarian cancer, Plinabulin promotes the polarization of TAMs toward an M1-like phenotype, which is associated with anti-tumor immunity. This M1 polarization leads to increased expression of pro-inflammatory markers (such as CD80 and CD86) and cytokines (like IL-1β, IL-6, and IL-12), while reducing levels of immunosuppressive cytokines such as IL-10. Through activation of the JNK pathway, Plinabulin not only encourages M1 polarization but also selectively boosts the proliferation of these M1-like macrophages. This shift reprograms TAMs from supporting tumor growth to actively fighting it, thereby enhancing the tumor’s vulnerability to immune attack. In co-culture experiments, plinabulin-polarized macrophages directly killed ovarian cancer cells via Fas/Fas-L signaling, demonstrating its potential as a powerful agent in anti-tumor immunotherapy (178). Similarly, erastin is a small molecule known for inducing ferroptosis, a form of cell death driven by iron-dependent lipid peroxidation, and has shown potential in cancer therapy. However, in ferroptosis-resistant OC cells, erastin paradoxically enhances metastatic potential by influencing the tumor microenvironment. Specifically, erastin promotes the M2 polarization of TAMs, a state associated with tumor progression. This effect occurs through activation of the STAT3 signaling pathway, which increases IL-8 secretion by macrophages. The elevated IL-8 levels, in turn, stimulate EMT in OC cells, facilitating invasion and migration. Blocking IL-8 or STAT3 disrupts this pathway, reducing the pro-metastatic effects induced by erastin-treated TAMs, thereby identifying the STAT3/IL-8 axis as a potential therapeutic target to counteract erastin’s unintended enhancement of metastasis in resistant OC cells (179). In addition, paclitaxel is a widely used chemotherapy drug that stabilizes microtubules, causing cell-cycle arrest in cancer cells. Beyond its cytotoxic effects, Paclitaxel has shown immune-modulatory properties, specifically reprogramming TAMs from a tumor-promoting M2 phenotype to an antitumor M1 phenotype through TLR4 (Toll-like receptor 4) signaling. In ovarian cancer, Paclitaxel’s interaction with TLR4 on TAMs activates NF-κB, shifting macrophages toward an M1 profile marked by increased production of pro-inflammatory cytokines such as TNFα and IL12. This M1 reprogramming decreases the immune-tolerant environment within tumors, reducing tumor progression and potentially enhancing the efficacy of immunotherapies. Paclitaxel’s impact on TAM polarization through TLR4 could be leveraged to strengthen antitumor immunity in ovarian cancer, offering a promising strategy for combination cancer therapies (180). In HGSOC, AZD5153, a BRD4 inhibitor, shows promising effects in reprogramming TAMs from an M2-like, immunosuppressive phenotype to an M1-like, pro-inflammatory phenotype. AZD5153 achieves this by inhibiting BRD4’s influence on MAF, a transcription factor crucial for M2 polarization. This shift increases pro-inflammatory cytokines, such as IL-12, and decreases immunosuppressive cytokines like IL-10, enhancing the immune response within the tumor microenvironment. Notably, AZD5153-activated TAMs improve CD8+ T cell activation, although not their proliferation, strengthening the antitumor immunity in HGSOC. Additionally, AZD5153 downregulates PD-L1 expression on TAMs, effectively sensitizing ovarian tumors to anti-PD-L1 therapies. This dual approach, direct tumor cell inhibition alongside immune modulation, positions AZD5153 as a potential enhancer for checkpoint inhibitor therapies, offering a promising combinatorial strategy to improve HGSOC patient outcomes (181). Digitoxin, a cardiac glycoside traditionally used to treat heart conditions, has shown potential as an anticancer agent by influencing TME in ovarian cancer. Although digitoxin does not alter macrophage polarization directly (meaning it does not shift macrophages between M1 and M2 phenotypes), it impacts macrophage-related processes within the TME. Specifically, digitoxin inhibits the migration and tube formation of endothelial cells and suppresses ovarian cancer cell migration and proliferation in response to signals from both M1- and M2-polarized macrophages. It achieves these effects by inhibiting focal adhesion kinase (FAK) phosphorylation, which is critical for cell motility and angiogenesis. This selective inhibition highlights digitoxin’s potential in targeting cancer cell interactions with the TME, without modifying the macrophage polarization itself, offering promise for its repositioning as an adjunct therapy in metastatic ovarian cancer (182). Lastly, BP1003 is a novel anticancer therapeutic that targets STAT3, a protein involved in promoting tumor growth, drug resistance, and immune evasion. It consists of a nuclease-resistant antisense oligodeoxynucleotide (ASO) encapsulated in a neutral liposome, designed to inhibit the expression of STAT3. In ovarian cancer, BP1003 has demonstrated significant anti-tumor effects by reducing STAT3 levels, which in turn decreases cell viability, migration, and spheroid formation, especially when combined with chemotherapy agents like paclitaxel and 5-fluorouracil. Additionally, BP1003 effectively disrupts the polarization of monocytes into M2 macrophages, which are typically associated with creating a pro-tumorigenic environment by suppressing immune responses. This selective inhibition of M2 macrophage polarization without affecting M1 macrophages, which are anti-tumorigenic, suggests that BP1003 has potential not only as a direct tumor inhibitor but also as an immunomodulatory agent, enhancing its overall therapeutic potential against ovarian cancer (183).

### Natural products

Natural products have shown potential to enhance the efficacy of immunotherapy in ovarian cancer by modulating the TME and supporting immune system function. Compounds derived from plants, marine organisms, and other natural sources, such as curcumin, resveratrol, and fucoidan, have demonstrated anti-inflammatory and immunomodulatory properties that may reduce immunosuppressive signals within the TME (184). These natural agents can inhibit regulatory T cells, myeloid-derived suppressor cells (MDSCs), and TAMs, thus alleviating immune suppression and allowing for a stronger anti-tumor immune response. Additionally, certain natural products enhance dendritic cell activity, T cell infiltration, and antigen presentation, making immunotherapy treatments, such as immune checkpoint inhibitors and CAR-T cells, more effective. By acting on both tumor cells and immune cells, these natural compounds can boost the overall effectiveness of immunotherapy and contribute to improved treatment outcomes in ovarian cancer (185).

#### STAT3 inhibitors

Cardamonin, a natural chalcone compound, demonstrates potential as an anti-cancer agent by targeting TAMs in ovarian cancer. TAMs, especially in their M2-polarized form, play a significant role in tumor progression by secreting pro-tumorigenic factors that support cancer growth, angiogenesis, and metastasis. In ovarian cancer, cardamonin suppresses M2 polarization and reduces TAM-mediated tumor support by inhibiting the mTOR signaling pathway and STAT3 activation. This inhibition decreases the expression of key tumor-promoting factors like IL-6, VEGFα, MMP2, and MMP9, limiting the tumor-promoting environment created by TAMs. Cardamonin’s impact on both mTOR and STAT3 pathways emphasizes its role as a potential therapeutic agent that could disrupt TAMs’ support of ovarian cancer, offering a promising avenue for enhancing immunotherapy in this challenging cancer type (186). Corosolic acid (CA), a natural compound known for its potent inhibitory effect on STAT3 signaling, shows promise as an adjunctive treatment for epithelial ovarian cancer (EOC) by enhancing the effects of chemotherapy and disrupting tumor-promoting interactions. In ovarian cancer cell lines (SKOV3, RMG-1, and ES-2), CA inhibited cell proliferation and increased apoptosis by downregulating STAT3, a key pathway associated with tumor growth, chemoresistance, and immune suppression. Furthermore, CA increased the efficacy of chemotherapy agents like paclitaxel and cisplatin by sensitizing EOC cells to these treatments through STAT3 suppression. Additionally, CA prevented the polarization of macrophages into the M2 phenotype, a macrophage subtype that supports tumor progression through STAT3 activation in cancer cells. By inhibiting this M2 polarization, CA reduced tumorigenic interactions in the ovarian cancer microenvironment. These findings highlight CA’s potential as an effective adjuvant for EOC, leveraging its dual action on both cancer cells and TAMs to improve treatment outcomes (187).

Onionin A (ONA), a sulfur-containing natural compound derived from onions, shows promising anti-ovarian cancer activity by targeting both cancer cells and TAMs. In studies involving ovarian cancer cell lines (SKOV3, ES2, RMG1) and a murine ovarian cancer model, ONA inhibited cancer cell proliferation by suppressing STAT3 activation, a pathway crucial to cancer progression and chemoresistance. ONA also interfered with the cell-cell interactions that enhance TAMs’ protumor functions, reducing the secretion of M2 macrophage markers like IL-10 and other tumor-promoting cytokines. Additionally, ONA showed synergistic effects with chemotherapy drugs such as cisplatin (CDDP) and paclitaxel (PTX), increasing cancer cell sensitivity and enabling potential dose reductions of these agents. *In vivo*, ONA administration not only reduced tumor growth and prolonged survival but also decreased the infiltration of pro-tumor CD163+ macrophages in tumor tissues. These findings suggest that ONA, as an orally available compound, could be an effective adjuvant in treating advanced ovarian cancer by targeting both cancer cells and their supportive macrophage environment (188).

#### PI3K/Akt/mTOR pathway inhibitors

Triptolide (TPL), a bioactive compound from Tripterygium wilfordii, has shown promise in treating drug-resistant ovarian cancer by targeting M2 TAMs and modifying the tumor microenvironment. In studies using A2780/DDP ovarian cancer cells, TPL reduced cell proliferation, invasion, and migration by inhibiting M2 macrophage polarization through the PI3K/AKT/NF-κB signaling pathway. This effect was even more pronounced when TPL was combined with cisplatin (DDP), which enhanced anti-tumor responses by decreasing markers of tumor vascularization (CD31) and M2 polarization (CD206), resulting in significant tumor growth inhibition and increased survival *in vivo*. Additionally, TPL and DDP together shifted the gut microbiota composition, increasing beneficial bacteria like Akkermansia and Clostridium while reducing pathogenic Sutterella and Adlercreutzia, suggesting an indirect immune-supportive mechanism. These findings support TPL as a potential adjunctive therapy in combating chemoresistant ovarian cancer, offering a novel approach that targets immune modulation and gut microbiota for enhanced therapeutic efficacy (189). Neferine, a natural compound from Nelumbo nucifera (lotus), exhibits anti-angiogenic properties and impacts TAMs in high-grade serous ovarian carcinoma (HGSOC), potentially aiding in treatment-resistant cases. By inhibiting the mTOR/p70S6K pathway, Neferine promotes autophagy in endothelial cells, arresting cell growth and reducing blood vessel formation. Additionally, Neferine prevents macrophage polarization into the M2 phenotype, which is known to support tumor angiogenesis and growth, as M2 macrophages release pro-angiogenic factors like VEGF. In studies, treatment with Neferine showed decreased vascular formation in ovarian cancer models and a notable reduction in CD206-positive M2 macrophages, which correlated with improved overall survival in patients. This dual action on autophagy induction and M2 macrophage inhibition highlights Neferine’s potential as a complementary agent in anti-angiogenesis therapies, especially in HGSOC cases unresponsive to standard treatments (190).

Deoxyschizandrin, a primary lignan extracted from Schisandra berries, has demonstrated notable anti-cancer properties, particularly against ovarian cancer. It functions by inducing cell cycle arrest in ovarian cancer cells, specifically halting growth at the G0/G1 phase, which is achieved through the suppression of cyclin E, a protein crucial for cell cycle progression. This arrest reduces cancer cell proliferation, which is further reinforced by increased reactive oxygen species (ROS) production that disrupts the PI3K/Akt pathway, a critical pathway for cell survival and proliferation in cancer. Additionally, Deoxyschizandrin significantly inhibits the protumoural activation of TAMs, which are often reprogrammed by the tumor environment to support cancer growth. By reducing the expression of M2 macrophage markers (CD163 and CD209) and suppressing the release of tumor-promoting factors such as MMP-9, RANTES, and VEGF, Deoxyschizandrin mitigates the supportive role of TAMs in tumor progression. These combined actions highlight Deoxyschizandrin’s potential as an agent to limit both cancer cell growth and the tumor-supportive microenvironment in ovarian cancer (191).

#### NF-κB pathway inhibitors

Kaempferia parviflora (KP) extract has shown promising anti-inflammatory and anti-cancer effects in ovarian clear cell carcinoma (OCCC), which often exhibits poor responses to standard chemotherapy. In OCCC TOV-21G cells, KP extract significantly inhibited the release of inflammatory cytokines such as IL-6 and MCP-1, particularly in the presence of TNF-α stimulation. This suppression was achieved through the inhibition of the NF-κB signaling pathway by reducing NF-κB’s nuclear translocation and upregulating the inhibitory protein IκB. Furthermore, KP extract decreased the phosphorylation of ERK1/2 and AKT, key components of cell growth and survival signaling, and reduced levels of the anti-apoptotic protein MCL-1, thus promoting apoptosis. The anti-inflammatory action of KP extract extended to inhibiting the migration of monocytes (THP-1 cells) toward the tumor microenvironment by lowering MCP-1 levels, suggesting a potential reduction in TAM infiltration. This indicates KP extract’s ability to disrupt inflammatory pathways and monocyte recruitment in OCCC, positioning it as a potential adjuvant to enhance conventional therapies and improve outcomes in chemoresistant ovarian cancer (192). Verbascoside (VB), a phenylpropanoid glycoside, demonstrates significant anti-tumor activity against ovarian cancer (OC) by facilitating M1 macrophage polarization through the CCN1-AKT/NF-κB signaling pathway. Studies on OC cell lines (SKOV3 and A2780) and xenograft mouse models showed that VB suppresses OC cell proliferation, migration, and promotes apoptosis. VB induces M1 polarization of macrophages, as evidenced by increased M1 markers (CD86, IL-6, CXCL10) and reduced M2 marker expression. Mechanistically, VB directly binds and downregulates CCN1, an oncogenic factor highly expressed in OC, inhibiting the AKT/NF-κB pathway, which plays a central role in regulating tumor progression and macrophage polarization. The effect of VB on promoting M1 polarization and suppressing OC progression is enhanced by AKT inhibition (using LY294002), but reduced by CCN1 overexpression. *In vivo* experiments confirm that VB reduces tumor volume and weight, inhibits cell proliferation, and promotes apoptosis in OC tissues, with CCN1 overexpression diminishing VB’s effects. These findings suggest VB as a promising therapeutic agent for OC, working by altering macrophage polarization and suppressing tumorigenic pathways through CCN1-mediated AKT/NF-κB inhibition. Further studies are required to confirm the detailed mechanism of VB in macrophage polarization and its clinical efficacy in OC treatment (193).

#### HMGB1-TLR4 pathway inhibitors

Astragaloside IV is a natural compound derived from Radix Astragali, a traditional Chinese herb, known for its anti-inflammatory and anticancer properties. In ovarian cancer, it has shown promise by targeting the tumor microenvironment, specifically by suppressing the polarization of macrophages into the M2 pro-tumor phenotype, a shift usually triggered by interleukin IL-4 and IL-13 exposure. M2 macrophages promote cancer cell growth, invasion, and metastasis by secreting factors like TGF-β and MMP-9, which enhance the tumor’s invasive potential. Astragaloside IV interferes with this process by inhibiting the HMGB1-TLR4 signaling pathway, which is integral to M2 macrophage polarization. By blocking this pathway, Astragaloside IV reduces the expression of M2 markers and decreases the release of pro-tumor cytokines, ultimately weakening the supportive role of M2 macrophages in cancer progression and metastasis, offering a potential adjunctive strategy in ovarian cancer therapy (194).

#### RhoA-YAP1 pathway modulators

Polyunsaturated fatty acids (PUFAs) significantly contribute to the protumor microenvironment in ovarian cancer by promoting the polarization and deposition of TAMs toward an M2-like, immunosuppressive phenotype. Within the lipid-rich ascites of ovarian cancer, PUFAs inhibit RhoA-GTPase activity, leading to the downregulation of nuclear YAP1, a critical transcription factor in the Hippo pathway, which dampens antitumor immune responses. This suppression of RhoA-YAP1 signaling reprograms macrophages toward a protumoral M2-like polarization, facilitating tumor growth and metastasis. Additionally, these PUFA-enriched TAMs exhibit heightened lipid-oxidative phosphorylation metabolism, aligning with an M2-like, immune-dampening functionality that hinders cytotoxic CD8+ T-cell infiltration, thereby impairing adaptive immune responses against the tumor. Notably, this pathway can be reversed by activating YAP1 through MST1/2 inhibition, which shifts macrophages from an M2 to an M1 phenotype, enhancing CD8+ T-cell activity and mitigating tumor progression. This pathway underscores the therapeutic potential of targeting RhoA-YAP1 signaling to modify TAM polarization and improve outcomes in ovarian cancer (195).

#### ROS-induced apoptosis and M1 macrophage activation

The ethanol extract of Solanum lyratum Thunb (SLT), a traditional Chinese medicinal herb, has demonstrated significant anticancer effects on ovarian cancer cells through its impact on cellular proliferation, apoptosis, and EMT. *In vitro* studies using ovarian cancer cell lines A2780 and SKOV3 reveal that SLT reduces cell viability by activating a ROS-mediated p53 pathway, resulting in G1 cell cycle arrest, enhanced apoptosis, and inhibition of cell migration and invasion. SLT also plays a crucial role in modulating the tumor immune microenvironment by influencing macrophage polarization: it promotes the shift of M0 macrophages toward the M1 phenotype, marked by increased CD86+ cells, while inhibiting the M2 phenotype, characterized by decreased CD206+ cells. This dual action not only suppresses tumor-promoting macrophage types but also enhances pro-inflammatory macrophages, which support anticancer immune responses. Overall, these findings suggest that SLT, through ROS accumulation and p53 pathway activation, could serve as a promising basis for developing new ovarian cancer treatments by directly inhibiting cancer progression and enhancing the tumor immune response (196).

9-Hydroxycanthin-6-one, a β-carboline alkaloid derived from the stem bark of Ailanthus altissima, exhibits significant anti-cancer effects against ovarian cancer cells by inducing apoptosis through caspase- and reactive oxygen species (ROS)-dependent pathways. The compound promotes apoptosis in cancer cells by activating key caspases (caspase-3, -8, and -9) and increasing intracellular ROS, which signals apoptotic cell death. Additionally, 9-hydroxycanthin-6-one plays a role in modulating the tumor microenvironment by inhibiting the recruitment and activation of TAMs. It reduces the expression of chemokines MCP-1 and RANTES, which are essential for TAM recruitment, and further suppresses TAM activation by decreasing M2 macrophage markers and tumor-promoting factors like MMP-2, MMP-9, and VEGF. This dual action, directly inducing cancer cell apoptosis and modulating TAM activity, highlights 9-hydroxycanthin-6-one’s potential as a therapeutic agent in ovarian cancer treatment (197).

### Engineered EVs

Macrophage-engineered vesicles (MEVs) are bioengineered nanovesicles derived from the membranes of macrophages, particularly those polarized to the M1, or pro-inflammatory, phenotype. MEVs are created through processes like nitrogen cavitation, where macrophage cell membranes are fragmented and reassembled into small, distinct vesicular units that retain the functional properties of their parent macrophages. MEVs are designed to act as both immune modulators and therapeutic delivery vehicles. The repolarization effect of M1 MEVs works by converting M2 macrophages, which are typically associated with tumor progression and immune suppression, into M1-like macrophages that promote an anti-tumor immune response (198, 199). When M1 MEVs are introduced into the tumor microenvironment, they influence the surrounding M2 macrophages to adopt a more pro-inflammatory, tumor-fighting state by increasing the secretion of cytokines such as TNF-α and upregulating pro-inflammatory markers like CXCL8. This repolarization disrupts the immunosuppressive environment that favors tumor growth, thereby enhancing the immune system’s ability to target and destroy cancer cells. In addition to their immunomodulatory effects, M1 MEVs demonstrate direct anti-cancer activity against ovarian cancer cells by reducing cell viability when co-cultured with cancer cells, as seen in various ovarian cancer models like Caov-3 and OVCAR3. Furthermore, M1 MEVs have shown the capability to localize specifically to tumor sites *in vivo*. In mouse models, fluorescently labeled M1 MEVs derived from the murine macrophage cell line RAW264.7 successfully homed to ovarian cancer tumor xenografts after intravenous or intraperitoneal administration. This selective tumor localization highlights their potential for targeted cancer therapy, making them a promising tool for delivering therapeutic agents directly to the tumor site, minimizing off-target effects and enhancing treatment efficacy (200). Umbilical cord-derived macrophage exosomes loaded with cisplatin (exoCIS) show significant potential in enhancing the treatment of ovarian cancer, especially in overcoming drug resistance. Exosomes from M1 macrophages, when loaded with cisplatin, increased the cytotoxicity of the drug by 3.3 times in drug-resistant ovarian cancer cells (A2780/DDP) and by 1.4 times in drug-sensitive cells (A2780) compared to cisplatin alone. M2 macrophage-derived exosomes also increased cytotoxicity, though less effectively than M1-derived exosomes. These exosomes are naturally better suited for drug delivery due to their stability, low immunogenicity, and ability to specifically accumulate in tumor cells. Moreover, the study highlighted that the method of sonication for loading cisplatin into exosomes provided greater efficiency and resulted in a more sustained drug release. Incorporating cisplatin into M1 exosomes not only reduced the IC50 (a measure of drug potency) but also induced higher rates of apoptosis in ovarian cancer cells, indicating that this method could help overcome platinum resistance in ovarian cancer therapy​ (201).

### Oncolytic viruses

Oncolytic virus (OV) therapy represents an innovative approach for treating ovarian cancer, primarily through viruses engineered to selectively infect and destroy cancer cells. The direct lysis of tumor cells triggers the release of tumor-specific antigens, which subsequently stimulate a robust anti-tumor immune response. This mechanism underpins the primary therapeutic role of OVs, while additional modulation of the tumor microenvironment (TME), including macrophage polarization, has emerged as an ancillary benefit (202–204). There is evidence suggesting that OVs can influence macrophage polarization, shifting pro-tumorigenic M2 macrophages toward an anti-tumorigenic M1 phenotype. For instance, oncolytic vaccinia viruses, like OncoVV-AVL, have been shown to increase levels of interferon-gamma (IFN-γ), a cytokine linked to M1 polarization and enhanced immune response. However, it is important to note that macrophage repolarization is not the primary mechanism of action for OVs, and the extent to which OVs alone affect macrophage polarization may be limited (205). Recent research has highlighted the potential for OVs to function synergistically with therapies that more directly target macrophages. Specifically, the use of CSF1R inhibitors has been reported to improve the efficacy of OVs by directly suppressing M2 macrophages and enhancing any macrophage-repolarizing effects OVs may initiate. Such findings suggest that OVs on their own may not efficiently remodel the immunosuppressive TME but can complement macrophage-targeted therapies or immune checkpoint inhibitors to achieve more significant therapeutic outcomes.

In ovarian cancer treatment, oncolytic virus therapy holds promise as a component of combination regimens that integrate direct macrophage-targeted therapies. While their immunomodulatory effects, including macrophage repolarization, contribute to their broader anti-tumor mechanism, future studies are needed to elucidate the specific pathways involved and to optimize strategies for combining OVs with macrophage-repolarizing therapies like CSF1R inhibitors to improve clinical outcomes (206).

## Conclusion and future perspectives

Ovarian cancer continues to be the deadliest among gynecological cancers, primarily due to its subtle beginnings, diagnosis at advanced stages, frequent relapses, and inherent resistance to standard chemotherapy treatments. A significant factor contributing to the complexity and aggressiveness of OC is the intricate dynamics within TME, especially the involvement of TAMs. Macrophage polarization, the process by which macrophages adopt either a pro-inflammatory (M1) or anti-inflammatory (M2) state, is crucial in influencing tumor growth, immune evasion, and resistance to therapies in ovarian cancer. This review highlights that the dominance of M2-like TAMs in the OC TME creates an environment that supports tumor survival, promotes angiogenesis, facilitates metastasis, and suppresses adaptive immune responses through the secretion of various pro-tumorigenic factors such as IL-10, TGF-β, and VEGF. The shift of macrophages toward the M2 phenotype is driven by numerous molecular regulators and signaling pathways, including the PI3K/AKT/mTOR, NF-κB, JAK/STAT, and Wnt/β-catenin pathways. Additionally, extracellular EVs and ncRNAs play vital roles in modulating macrophage behavior, further reinforcing the immunosuppressive environment of OC. A consistent finding across multiple studies is the identification of key molecular targets that influence macrophage polarization. Factors like PTEN loss, activation of Wnt/β-catenin signaling, and overexpression of proteins such as CBX2 and HE4 are frequently linked to enhancing the M2 phenotype and poorer clinical outcomes. On the other hand, strategies aimed at reprogramming TAMs toward the M1 phenotype, using agents like oncolytic viruses and engineered nanoparticles, have shown promise in reversing immunosuppression and improving the effectiveness of immunotherapies. Natural compounds, including cardamonin and curcumin, also emerge as potential modulators of macrophage polarization, presenting opportunities for adjunctive therapies that complement existing treatment methods. Moreover, the interaction between ovarian cancer stem cells and TAMs, mediated through signaling pathways like STAT3 and Wnt, underscores the necessity for comprehensive therapeutic approaches that address both cellular and molecular aspects of tumor biology. This reciprocal relationship enhances tumor survival, resistance to treatment, and aggressiveness, highlighting the need for strategies that can disrupt these interactions effectively. Despite advancements in understanding macrophage polarization in OC, several challenges persist. Early detection of ovarian cancer remains difficult, and the heterogeneous nature of TAMs across different tumor sites complicates the development of universally effective therapies. Additionally, the plasticity of macrophages, which allows them to switch phenotypes in response to environmental changes, poses a challenge for therapeutic interventions aimed at reprogramming them.

## Future perspectives

Looking forward, future research should focus on several key areas to advance the therapeutic landscape for ovarian cancer:

- Targeted Reprogramming of TAMs: Developing more precise and effective methods to shift TAMs from the M2 to the M1 phenotype is essential. This includes advancing nanoparticle-based delivery systems and engineered EVs that can specifically target TAMs and modulate their behavior without affecting other immune cells.
- Combination Therapies: Integrating TAM-targeting agents with existing immunotherapies, such as immune checkpoint inhibitors and CAR-T cell therapies, could synergistically enhance anti-tumor immune responses and overcome resistance mechanisms.
- Biomarker Development: Identifying robust biomarkers related to macrophage polarization will be crucial for patient stratification and monitoring therapeutic responses. Markers like CD163, LILRB1, and MUC2 have shown promise and require further validation in clinical settings.
- Understanding TAM Heterogeneity: Delving deeper into the diversity of TAMs within different ovarian cancer subtypes and metastatic sites will provide insights necessary for more tailored and effective therapeutic strategies.
- Exploration of Natural Compounds: Further investigation into natural products at the clinical level that influence macrophage polarization offers a complementary approach to conventional therapies, potentially reducing toxicity and enhancing efficacy.
- Clinical Trials: Translating preclinical findings into clinical applications through well-designed clinical trials is essential to validate the efficacy and safety of TAM-targeting therapies in ovarian cancer patients.
- Mechanistic Studies: Comprehensive studies to elucidate the underlying mechanisms by which various signaling pathways and molecular factors influence macrophage polarization will aid in identifying novel therapeutic targets.
- TAMs heterogeneity: Given the significant heterogeneity of TAMs in ovarian cancer, future therapeutic strategies must consider context-specific targeting of macrophage subpopulations. Approaches such as single-cell transcriptomic-guided therapies may enable precision targeting of immunosuppressive TAM subsets while preserving or enhancing the function of pro-inflammatory macrophages. Additionally, interventions designed to alter macrophage metabolism, such as lactate blockade or glutamine inhibition, may reprogram TAMs within specific tumor niches. Therapies aimed at macrophage-TME interactions, such as inhibiting macrophage-derived EV signaling, represent another promising avenue. By incorporating a nuanced understanding of TAM heterogeneity, emerging therapies may overcome the limitations of broad-spectrum macrophage-targeting approaches, improving clinical outcomes in ovarian cancer.

In summary, macrophage polarization is a critical determinant of ovarian cancer progression and therapy resistance. By continuing to unravel the complex molecular interactions within the TME and developing innovative strategies to manipulate TAM behavior, there is significant potential to improve treatment outcomes and survival rates for patients afflicted with this formidable disease.
